# Facilitating safe discharge through predicting disease progression in moderate COVID-19: a prospective cohort study to develop and validate a clinical prediction model in resource-limited settings

**DOI:** 10.1093/cid/ciac224

**Published:** 2022-03-21

**Authors:** Arjun Chandna, Raman Mahajan, Priyanka Gautam, Lazaro Mwandigha, Karthik Gunasekaran, Divendu Bhusan, Arthur T L Cheung, Nicholas Day, Sabine Dittrich, Arjen Dondorp, Tulasi Geevar, Srinivasa R Ghattamaneni, Samreen Hussain, Carolina Jimenez, Rohini Karthikeyan, Sanjeev Kumar, Shiril Kumar, Vikash Kumar, Debasree Kundu, Ankita Lakshmanan, Abi Manesh, Chonticha Menggred, Mahesh Moorthy, Jennifer Osborn, Melissa Richard-Greenblatt, Sadhana Sharma, Veena K Singh, Vikash K Singh, Javvad Suri, Shuichi Suzuki, Jaruwan Tubprasert, Paul Turner, Annavi M G Villanueva, Naomi Waithira, Pragya Kumar, George M Varghese, Constantinos Koshiaris, Yoel Lubell, Sakib Burza

**Affiliations:** Cambodia Oxford Medical Research Unit, Angkor Hospital for Children, Siem Reap, Cambodia; Centre for Tropical Medicine & Global Health, University of Oxford, Oxford, UK; Médecins Sans Frontières, New Delhi, India; Department of Infectious Diseases, Christian Medical College, Vellore, India; Nuffield Department of Primary Care Health Sciences, University of Oxford, Oxford, UK; Department of Medicine, Christian Medical College, Vellore, India; Department of Internal Medicine, All India Institute of Medical Sciences, Patna, India; Centre for Tropical Medicine & Global Health, University of Oxford, Oxford, UK; Mahidol Oxford Tropical Medicine Research Unit, Mahidol University, Bangkok, Thailand; Centre for Tropical Medicine & Global Health, University of Oxford, Oxford, UK; Mahidol Oxford Tropical Medicine Research Unit, Mahidol University, Bangkok, Thailand; Centre for Tropical Medicine & Global Health, University of Oxford, Oxford, UK; Foundation for Innovative Diagnostics, Geneva, Switzerland; Centre for Tropical Medicine & Global Health, University of Oxford, Oxford, UK; Mahidol Oxford Tropical Medicine Research Unit, Mahidol University, Bangkok, Thailand; Department of Transfusion Medicine & Immunohaematology, Christian Medical College, Vellore, India; Médecins Sans Frontières, New Delhi, India; Médecins Sans Frontières, New Delhi, India; Médecins Sans Frontières, New Delhi, India; Department of Infectious Diseases, Christian Medical College, Vellore, India; Department of Cardiothoracic & Vascular Surgery, All India Institute of Medical Sciences, Patna, India; Department of Virology, Rajendra Memorial Research Institute of Medical Sciences, Patna, India; Médecins Sans Frontières, New Delhi, India; Department of Infectious Diseases, Christian Medical College, Vellore, India; Médecins Sans Frontières, New Delhi, India; Department of Infectious Diseases, Christian Medical College, Vellore, India; Mahidol Oxford Tropical Medicine Research Unit, Mahidol University, Bangkok, Thailand; Department of Clinical Virology, Christian Medical College, Vellore, India; Foundation for Innovative Diagnostics, Geneva, Switzerland; Perelman School of Medicine, University of Pennsylvania, Philadelphia, USA; Department of Biochemistry, All India Institute of Medical Sciences, Patna, India; Department of Burns & Plastic Surgery, All India Institute of Medical Sciences, Patna, India; Médecins Sans Frontières, New Delhi, India; Médecins Sans Frontières, New Delhi, India; School of Tropical Medicine & Global Health, Nagasaki University, Nagasaki, Japan; Mahidol Oxford Tropical Medicine Research Unit, Mahidol University, Bangkok, Thailand; Cambodia Oxford Medical Research Unit, Angkor Hospital for Children, Siem Reap, Cambodia; Centre for Tropical Medicine & Global Health, University of Oxford, Oxford, UK; School of Tropical Medicine & Global Health, Nagasaki University, Nagasaki, Japan; Centre for Tropical Medicine & Global Health, University of Oxford, Oxford, UK; Mahidol Oxford Tropical Medicine Research Unit, Mahidol University, Bangkok, Thailand; Department of Community & Family Medicine, All India Institute of Medical Sciences, Patna, India; Department of Infectious Diseases, Christian Medical College, Vellore, India; Nuffield Department of Primary Care Health Sciences, University of Oxford, Oxford, UK; Centre for Tropical Medicine & Global Health, University of Oxford, Oxford, UK; Mahidol Oxford Tropical Medicine Research Unit, Mahidol University, Bangkok, Thailand; Médecins Sans Frontières, New Delhi, India; Department of Clinical Research, London School of Hygiene & Tropical Medicine, London, UK

**Keywords:** COVID-19, prognostic model, triage, low- and middle-income country, LMIC

## Abstract

**Background:**

In locations where few people have received COVID-19 vaccines, health systems remain vulnerable to surges in SARS-CoV-2 infections. Tools to identify patients suitable for community-based management are urgently needed.

**Methods:**

We prospectively recruited adults presenting to two hospitals in India with moderate symptoms of laboratory-confirmed COVID-19 in order to develop and validate a clinical prediction model to rule-out progression to supplemental oxygen requirement. The primary outcome was defined as any of the following: SpO_2_ < 94%; respiratory rate > 30 bpm; SpO_2_/FiO_2_ < 400; or death. We specified *a priori* that each model would contain three clinical parameters (age, sex and SpO_2_) and one of seven shortlisted biochemical biomarkers measurable using commercially-available rapid tests (CRP, D-dimer, IL-6, NLR, PCT, sTREM-1 or suPAR), to ensure the models would be suitable for resource-limited settings. We evaluated discrimination, calibration and clinical utility of the models in a held-out temporal external validation cohort.

**Results:**

426 participants were recruited, of whom 89 (21.0%) met the primary outcome. 257 participants comprised the development cohort and 166 comprised the validation cohort. The three models containing NLR, suPAR or IL-6 demonstrated promising discrimination (c-statistics: 0.72 to 0.74) and calibration (calibration slopes: 1.01 to 1.05) in the validation cohort, and provided greater utility than a model containing the clinical parameters alone.

**Conclusions:**

We present three clinical prediction models that could help clinicians identify patients with moderate COVID-19 suitable for community-based management. The models are readily implementable and of particular relevance for locations with limited resources.

## AUTHORS

Arjun Chandna,[1,2] Raman Mahajan,[3] Priyanka Gautam,[4] Lazaro Mwandigha,[5] Karthik Gunasekaran,[6] Divendu Bhusan,[7] Arthur T L Cheung,[2,8] Nicholas Day,[2,8] Sabine Dittrich,[2,9] Arjen Dondorp,[2,8] Tulasi Geevar,[10] Srinivasa R Ghattamaneni,[3] Samreen Hussain,[3] Carolina Jimenez,[3] Rohini Karthikeyan,[4] Sanjeev Kumar,[11] Shiril Kumar,[12] Vikash Kumar,[3] Debasree Kundu,[4] Ankita Lakshmanan,[3] Abi Manesh,[4] Chonticha Menggred,[8] Mahesh Moorthy,[13] Jennifer Osborn,[9] Melissa Richard-Greenblatt,[14] Sadhana Sharma,[15] Veena K Singh,[16] Vikash K Singh,[3] Javvad Suri,[3] Shuichi Suzuki,[17] Jaruwan Tubprasert,[8] Paul Turner,[1,2] Annavi M G Villanueva,[17] Naomi Waithira,[2,8] Pragya Kumar,[18] George M Varghese,[4] Constantinos Koshiaris,[5] Yoel Lubell ,[2,8] and Sakib Burza[3,19]

## AFFILIATIONS

1. Cambodia Oxford Medical Research Unit, Angkor Hospital for Children, Siem Reap, Cambodia
2. Centre for Tropical Medicine & Global Health, University of Oxford, Oxford, UK
3. Médecins Sans Frontières, New Delhi, India
4. Department of Infectious Diseases, Christian Medical College, Vellore, India
5. Nuffield Department of Primary Care Health Sciences, University of Oxford, Oxford, UK

6. Department of Medicine, Christian Medical College, Vellore, India
7. Department of Internal Medicine, All India Institute of Medical Sciences, Patna, India
8. Mahidol Oxford Tropical Medicine Research Unit, Mahidol University, Bangkok, Thailand
9. Foundation for Innovative Diagnostics, Geneva, Switzerland
10. Department of Transfusion Medicine & Immunohaematology, Christian Medical College, Vellore, India
11. Department of Cardiothoracic & Vascular Surgery, All India Institute of Medical Sciences, Patna, India
12. Department of Virology, Rajendra Memorial Research Institute of Medical Sciences, Patna, India
13. Department of Clinical Virology, Christian Medical College, Vellore, India
14. Perelman School of Medicine, University of Pennsylvania, Philadelphia, USA
15. Department of Biochemistry, All India Institute of Medical Sciences, Patna, India
16. Department of Burns & Plastic Surgery, All India Institute of Medical Sciences, Patna, India
17. School of Tropical Medicine & Global Health, Nagasaki University, Nagasaki, Japan
18. Department of Community & Family Medicine, All India Institute of Medical Sciences, Patna, India
19. Department of Clinical Research, London School of Hygiene & Tropical Medicine, London, UK

Dr. Arjun Chandna, Cambodia Oxford Medical Research Unit, Siem Reap, Cambodia

We report three clinical prediction models to help identify which patients with moderate COVID-19 can be safely managed in the community. Each model contains three easily ascertained clinical parameters and one biochemical biomarker, measurable with commercially-available rapid tests.

*Background*

In locations where few people have received COVID-19 vaccines, health systems remain vulnerable to surges in SARS-CoV-2 infections. Tools to identify patients suitable for community-based management are urgently needed.

*Methods*

We prospectively recruited adults presenting to two hospitals in India with moderate symptoms of laboratory-confirmed COVID-19 in order to develop and validate a clinical prediction model to rule-out progression to supplemental oxygen requirement. The primary outcome was defined as any of the following: $SpO_2 < 94\%$; respiratory rate > 30 bpm; $SpO_2/FiO_2 < 400$; or death. We specified *a priori* that each model would contain three clinical parameters (age, sex and $SpO_2$) and one of seven shortlisted biochemical biomarkers measurable using commercially-available rapid tests (CRP, D-dimer, IL-6, NLR, PCT, sTREM-1 or suPAR), to ensure the models would be suitable for resource-limited settings. We evaluated discrimination, calibration and clinical utility of the models in a held-out temporal external validation cohort.

*Results*

426 participants were recruited, of whom 89 (21.0%) met the primary outcome. 257 participants comprised the development cohort and 166 comprised the validation cohort. The three models containing NLR, suPAR or IL-6 demonstrated promising discrimination (c-statistics: 0.72 to 0.74) and calibration (calibration slopes: 1.01 to 1.05) in the validation cohort, and provided greater utility than a model containing the clinical parameters alone.

*Conclusions*

We present three clinical prediction models that could help clinicians identify patients with moderate COVID-19 suitable for community-based management. The models are readily implementable and of particular relevance for locations with limited resources.

COVID-19; prognostic model; triage; low- and middle-income country; LMIC

## INTRODUCTION

In low-income countries, where fewer than 5% of people have received a COVID-19 vaccine,[1] fragile healthcare systems remain vulnerable to being overwhelmed by a surge in COVID-19 cases (Figure 1).[2-4]

A minority of patients with COVID-19 require admission to hospital. Oxygen is the most important supportive treatment and in most low- and middle-income countries (LMICs) is the practical ceiling of care.[5] The World Health Organization (WHO) estimates that 15% of patients with symptomatic COVID-19 will require supplemental oxygen.[6] Effective identification of patients who are unlikely to become hypoxic would have considerable benefit; tools to support triage could decompress healthcare systems by giving practitioners confidence to allocate resources more efficiently.[7]

Numerous prognostic models for COVID-19 have been developed.[8,9] Almost all predict critical illness or mortality and thus cannot inform whether a patient might be safely managed in the community. Of those that focus on patients with moderate disease, most rely on retrospective or registry-based data,[10-14] lack external validation,[15,16] and are not feasible for use in resource-limited settings.[9,17] Moreover, most existing studies did not follow best-practice guidelines for model building and reporting,[18] are at high risk of bias,[8] and the resulting models are neither suitable nor recommended for use in LMIC contexts.[9]

We set out to develop and validate a clinical prediction model to rule-out progression to supplemental oxygen requirement in patients presenting with moderate COVID-19. We hypothesised that combining simple clinical parameters with host biomarkers feasible for measurement in resource-limited settings and implicated in the pathogenesis of COVID-19 would improve prognostication.

## METHODS

*Study population*

PRIORITISE is a prospective observational cohort study. Consecutive patients aged ≥ 18 years with clinically-suspected SARS-CoV-2 infection presenting with moderate symptoms to the All India Institute of Medical Sciences (AIIMS) Hospital in Patna, India and the Christian Medical College (CMC) Hospital in Vellore, India were screened (daytime hours, Monday to Saturday). AIIMS is a 1,000-bed hospital and the largest medical facility providing primary-to-tertiary healthcare in the state of Bihar. CMC is a 3,000-bed not-for-profit hospital that provided care for ~1,500 patients with COVID-19 each day during the peak of the delta-wave surge in India.

We adapted the case definitions in the WHO Clinical Management guideline (moderate disease)[6] and WHO Clinical Progression Scale (WHO-CPS; scores 2, 3 or 4)[19] to define moderate disease as follows: a peripheral oxygen saturation ($SpO_2$) ≥ 94% and respiratory rate < 30 breaths per minute (bpm), in the context of systemic symptoms (breathlessness or fever and chest pain, abdominal pain, diarrhoea or severe myalgia), recognising that the threshold for hospitalisation varies throughout a pandemic and that a sensitive cut-off for hypoxia would be desirable in a tool to inform community-based management.[19,20]

*Data collection*

Structured case-report forms (appendix p2-10) were completed at enrolment, day 7, and day 14, and daily during admission to the study facilities. Anthropometrics and vital signs were measured at enrolment and demographics, clinical symptoms, comorbidities, and medication history collected via brief interview with the participant. Venous blood samples were collected at enrolment in ethylenediaminetetraacetic acid (EDTA) tubes. Participants were followed-up in-person whilst admitted to the facility, and by telephone on days 7 and 14 if discharged prior to this. Those discharged who reported worsening symptoms on day 7 and/or persistent symptoms on day 14 were recalled to have their $SpO_2$ and respiratory rate measured.

*Primary outcome*

The primary outcome was development of an oxygen requirement within 14 days of enrolment, defined as any of the following: $SpO_2 < 94\%$; respiratory rate > 30 bpm; $SpO_2/FiO_2 < 400$;[21,22] or death, aligning closely with a WHO-CPS score of ≥ 5.[19] Patients who received supplemental oxygen outside the study facilities were classified as meeting the primary outcome if it was not possible to retrieve their case notes, provided the oxygen was prescribed in a licensed medical facility. The site study teams were unaware of which baseline variables had been preselected as candidate predictors when determining outcome status.

*Candidate predictors*

We decided *a-priori* that a model using four predictors would be practical in high-patient-throughput resource-limited settings. Considering resource constraints, reliability, validity, feasibility, and biological plausibility, we prespecified that each model would contain age, sex, $SpO_2$ and one biochemical biomarker.[10,17,23]

Following a literature review (Figures S1-2), biomarkers were shortlisted in consultation with FIND, the global alliance for diagnostics (Geneva, Switzerland). To qualify for inclusion, biomarkers had to be quantifiable with rapid tests in clinical use or late-stage development (Technology Readiness Level ≥ 4; Table S1).[24] The final list included: C-reactive protein (CRP), D-dimer, interleukin-6 (IL-6), neutrophil-to-lymphocyte ratio (NLR), procalcitonin (PCT), soluble triggering receptor expressed on myeloid cells-1 (sTREM-1), and soluble urokinase plasminogen activator receptor (suPAR).[25-29]

Clinical predictors were measured at enrolment and all biomarkers except NLR were measured retrospectively from samples obtained at enrolment. NLR was measured on site and was not repeated if it had been measured at the site within 24 hours prior to recruitment. All predictors were measured blinded to outcome status.

*Laboratory procedures*

Complete blood counts (XP-300-Hematology-Analyzer, Sysmex, IL) were performed on site and aliquots of EDTA-plasma stored at -20°C or below until testing. Biomarker concentrations were quantified using the suPARnostic ELISA (ViroGates, Denmark) and Simple Plex Ella microfluidic platform (ProteinSimple, CA) as described elsewhere.[30] Remaining plasma was biobanked on site. SARS-CoV-2 IgG and IgM antibodies were measured using the SCoV-2 Detect ELISA (InBios, WA). Oral and/or nasopharyngeal swabs were collected to confirm SARS-CoV-2 infection via RT-PCR (Cepheid Xpert Xpress SARS-CoV-2, CA or Altona RealStar SARS-CoV-2 rRT-PCR, Germany).

*Sample size*

We considered the sample size for model development and validation separately. We followed the recommendations of Riley et al. and assumed a conservative $R^2$ Nagelkerke of 0.15.[31] We anticipated that ~8% of participants would meet the primary endpoint and estimated that 44 outcome events would be required to derive a prediction model comprising four candidate predictors and minimise the risk of overfitting (events per parameter [EPP] = 11).

Given the uncertainty around deterioration rates amongst patients with moderate COVID-19 at the time of study inception, we prespecified an interim review after the first 100 participants were recruited. At this review, the proportion of participants meeting the primary endpoint was higher than anticipated (20% vs. 8%). At this higher prevalence, and using $R^2$ values from 0.20-0.15, between 52-68 outcome events (EPP = 13-17) would be required to develop the prediction models.[31] Recognising that (i) our range of $R^2$ estimates was conservative, (ii) penalised regression methods would reduce the risk of overfitting, and (iii) the external validation cohort would allow assessment of model optimism, and following the advice of the External Advisory Panel, a decision was made to use the first 50 outcome events to derive the models. Participants recruited after that point were entered into the external temporal validation cohort.

*Model development and validation*

We explored the relationship between candidate predictors and the primary outcome using a Lowess smoothing approach to identify non-linear patterns.  Transformations were used when serious violations of linearity were detected. We used penalised logistic (ridge) regression to develop the models and shrink regression coefficients to minimise model optimism. All predictors were prespecified and no predictor selection was performed during model development. Due to few missing data (< 3% for any single predictor), missing observations were replaced with the median value, grouped by outcome status. A sensitivity analysis was conducted using full-case analysis.

We assessed discrimination (c-statistics) and calibration (calibration plots and slopes) for each model in the validation cohort, and examined classifications (true positives [TP], false positives [FP], true negatives [TN], false negatives [FN]) at clinically-relevant cut-points (predicted probabilities). Finally, recognising that the relative value of a TP and FP will vary at different stages of the pandemic,[20] we examined the potential clinical utility of the models using decision curve analyses to quantify the net benefit between correctly identified TP or TN and incorrectly identified FP or FN at a range of plausible trade-offs (threshold probabilities).[32]

All analyses were done in R v4.03.

*Ethical approvals*

This investigator-initiated study was prospectively registered (ClinicalTrials.gov; NCT04441372), with protocol and statistical analysis plan uploaded to the Open Science Framework platform (DOI: 10.17605/OSF.IO/DXQ43). Ethical approval was given by the AIIMS, Patna Ethics Committee; CMC Ethics Committee; Oxford Tropical Research Ethics Committee; and MSF Ethical Review Board.

## RESULTS

Between 22 October 2020 and 3 July 2021, 2,808 patients with clinically-suspected COVID-19 were screened, of whom 446 were eligible (446/2,808; 15.9%) and 426 were recruited (20/446; 4.5% refusal rate). Three participants were lost-to-follow-up (3/426; 0.7%) and excluded from further analyses (Figure 2). All participants had laboratory-confirmed SARS-CoV-2 infection (421/423 [99.5%] via RT-PCR). The maximum amount of missing data for any predictor was 2.6% (NLR; 11/423; Table S2). The first 257 participants comprised the development cohort and the remaining 166 participants comprised the temporal validation cohort.

*Outcomes*

Development and validation cohorts were largely balanced with respect to baseline characteristics (Table 1; Table S3). There was a higher proportion of males in the development cohort (72% [185/257] vs. 61% [101/166]). In the validation cohort, more participants had a qSOFA score ≥ 2 (16/166 [9.6%] vs. 13/257 [5.1%]), and the validation cohort had higher median CRP (58.1 mg/l vs. 24.4 mg/l) and IL-6 (31.6 pg/ml vs. 11.0 pg/ml) concentrations. Eighty-nine participants met the primary outcome (89/423; 21.0%); 50 in the development cohort (50/257; 19.5%) and 39 in the validation cohort (39/166; 23.5%). Median (IQR) time to oxygen requirement was 1 (1-3) day; 11 participants died, 2 were mechanically ventilated, 15 received non-invasive ventilation, 49 received oxygen via a face mask and/or nasal cannula (one outside the study facilities), and 12 had an $SpO_2 <$ 94% but did not receive oxygen supplementation (Table S4; Figure S3).

Relationships between candidate predictors and the primary outcome are illustrated (Figure S4), and c-statistics (continuous predictors) and odds ratios (continuous and categorical predictors) reported (Table S5). The full models are presented in the appendix (Table S5; Figure S5). After adjustment for the three clinical variables, five biomarkers (CRP, D-dimer, IL-6, NLR, and suPAR) were independently associated with development of an oxygen requirement.

*Prognostic models*

Discrimination and calibration of each model in the validation cohort are presented in Figure 3. C-statistics ranged from 0.66 (clinical model and model containing PCT) to 0.74 (model containing IL-6). Calibration slopes ranged from 0.62 (model containing PCT) to 1.01 (model containing suPAR). Calibration was better at lower predicted probabilities, with some models overestimating risk at higher predicted probabilities.

The ability of each model to rule-out progression to oxygen requirement amongst patients with moderate COVID-19 at predicted probabilities (cut-offs) of 10%, 15% and 20% is shown (Table 2; Table S6; Figure S6). A cut-off of 10% reflects a management strategy equivalent to admitting any patient in whom the predicted risk of developing an oxygen requirement is ≥ 10%. At this cut-off, the results suggest that a model containing the three clinical parameters (age, sex, and $SpO_2$) without any biomarkers could facilitate correctly sending home ~25% of patients with moderate COVID-19 who would not subsequently require supplemental oxygen, at the cost of also sending home ~9% of moderate patients who would deteriorate and require supplemental oxygen, i.e. a ratio of correctly to incorrectly discharged patients of 10:1.

The inclusion of either NLR or suPAR improved the predictive performance such that the ratio of correctly to incorrectly discharged patients increased to 23:1 or 25:1 respectively, whilst a model containing IL-6 resulted in a similar proportion (~21%) of correctly discharged patients as the clinical model but without missing any patients who would deteriorate and require supplemental oxygen. Inclusion of the other candidate biomarkers (CRP, D-dimer, PCT or sTREM-1) did not improve the ability of the clinical model to rule-out progression to supplemental oxygen requirement.

*Generalisability*

We recognised that the relative value of a TP and FP, i.e. admitted patients who would and would not subsequently require supplemental oxygen, was not fixed and would vary at different stages of the pandemic, reflecting bed pressures and/or capacity for follow-up.[20] Decision curve analyses accounting for this differential weighting suggest that the clinical model could provide utility (net benefit over an "admit-all" approach) at a threshold probability above 15% (i.e. when the value of one TP is equal to ~7 FPs). Furthermore, the results indicate that models containing any one of IL-6, NLR or suPAR could offer greater net benefit than the clinical model and extend the range of contexts in which a model might provide utility to include threshold probabilities above 5% (value of one TP is equal to 19 FPs; i.e. when bed pressures are less critical). For the model containing IL-6, this higher net benefit appeared to be maintained across a range of plausible threshold probabilities (Figure 4).

**DISCUSSION**

We report the development and temporal validation of three promising clinical prediction models to assist with the assessment of patients with moderate COVID-19. The models combine three simple parameters (age, sex, and $SpO_2$) with measurement of a single biochemical biomarker (IL-6, NLR or suPAR), quantifiable using commercially-available rapid tests.

We included patients in whom there is clinical uncertainty as to whether admission is warranted, and adopted an analytical approach which acknowledged that the trade-offs inherent in this decision will vary at different stages of the pandemic and in different healthcare settings. We used specific systemic symptoms to define moderate severity disease rather than the WHO-CPS, recognising, as did the scale's original authors, that the lower-end of the WHO-CPS is subjective.[19] Performance of any prediction model is sensitive to the prevalence of the outcome it aims to predict and thus we hope our more objective study entry criteria will better standardise the outcome prevalence and facilitate model transportability; we followed the widely-used ISARIC case report form to define symptoms to permit validation by other groups.[33]

Our approach focussed on quantifying the added value of host biomarkers. We recognise that laboratory tests carry an opportunity cost, especially when resources are limited. Although a model containing clinical parameters alone would be simpler to implement, our analyses indicate that inclusion of one biomarker test would allow use of the model in a broader range of contexts, including when bed pressures are less acute early in a COVID-19 surge.

Our models have face validity. All clinical and laboratory predictors have been implicated in the pathogenesis of COVID-19.[10,17,23,25,27,29] Similar to others, we found that age and sex were not strongly associated with risk of deterioration, in contrast to their well-recognised association with COVID-19 mortality.[23] This underlines the importance of developing models for specific clinical use-cases. Models developed to predict mortality are not necessarily appropriate to rule-out less severe disease, just as models developed in well-resourced healthcare systems may not generalise to resource-limited settings.[34]

The three biochemical biomarkers that demonstrate most promise in our study have biological plausibility. In addition to being a therapeutic target,[35] raised IL-6 levels predict development of an oxygen requirement,[27,28] and along with an elevated NLR, form part of the COVID-19-associated hyperinflammatory syndrome (cHIS) diagnostic criteria.[36] Elevated suPAR levels are associated with disease severity and progression in both moderate and severe COVID-19,[29,37] and have been used for stratification into trials of immunomodulatory agents.[38]

We addressed the limitations identified in other COVID-19 prognostic models by following the TRIPOD guidelines,[18] and using a prospectively collected dataset with minimal loss-to-follow-up and missing data.[8] Nevertheless, the small validation cohort (determined by the natural history of the pandemic in India) limits our ability to draw strong conclusions. Although the same models appeared superior in the different analyses we performed, further external validation is required before they can be recommended for use; we have published our full models (Table S5; Figure S5) to encourage independent validation.

No vaccinated individuals were included in the study. The models may require recalibration for use in vaccinated populations with lower baseline risk of progression to severe COVID-19. However, it is important to note that only 15/54 African countries met the WHO target of vaccinating 10% of their population by the end of September 2021.[40] An estimated 55-70% vaccination coverage is required to achieve herd immunity for a vaccine with 90% efficacy.[41] Unfortunately, the timelines for adequate vaccination coverage in many LMICs are likely to be long.

In our context, corticosteroids were readily available and often self-prescribed or used off-license. Although steroid use was associated with some candidate predictors, it was not associated with the primary outcome and is therefore unlikely to have confounded the observed association (Tables S7-8).

We selected oxygen requirement as our primary outcome as this reflects a clinically meaningful endpoint. We opted to use an $SpO_2/FiO_2 < 400$ for participants without documented hypoxia or tachypnoea prior to initiation of supplemental oxygen, as the threshold for oxygen therapy can be subjective and vary depending on available resources.[19,22] It is unlikely that our outcome lacked sensitivity; only one participant who received supplemental oxygen did not meet the primary outcome. It may have lacked specificity (12 participants who met the primary outcome did not receive supplemental oxygen and calculation of $FiO_2$ in non-ventilated patients can overestimate pulmonary dysfunction),[42] but sensitivity would always be prioritised in a tool to inform community-based management. Furthermore, any outcome misclassification is likely to have reduced, rather than exaggerated, the prognostic performance of the candidate predictors and models.[43]

Baseline Ct value was not associated with the risk of deterioration (Table S9). In keeping with others, we found that seronegativity at enrolment was associated with an increased risk of deterioration (49/190 [25.8%] vs. 37/222 [16.7%]; $X^2 = 5.16$; $p = 0.023$).[44,45] As rapid antibody tests are available this warrants further exploration, acknowledging that this is likely most relevant in patients without a history of previous COVID-19 illness or vaccination.

In conclusion, we present three clinical prediction models that could help clinicians to identify patients with moderate COVID-19 whom are suitable for community-based management. The models address an unmet need in the COVID-19 care continuum. They are of particular relevance where resources are scarce and, if validated, would be practical for implementation. Routinely collected data from MSF medical facilities across 26 LMICs indicate that 54.4% (18,400 / 33,780) of patients presenting with clinically-suspected COVID-19 between March 2020 and November 2021 whom might be considered for admission, or 16.2% of all patients (18,400 / 113,455), would have been eligible for assessment using our models, illustrating the potential for widespread impact.

## NOTES

## CONTRIBUTORS

AC, ND, AD, CJ, PT, GMV, YL, and SB conceptualised the study. ATLC conducted the biomarker literature review. AC, SD, JO, YL, and SB shortlisted the candidate biomarkers. PG, KG, DB, RK, SK, AL, AM, VeKS, JS, and PK collected the clinical data. TG, ShK, VK, DK, MM, MRG, SaS, ViKS, and SB were responsible for specimen processing and the laboratory assays. AC, RM, PG, LM, SRG, SH, CM, and NW curated the data. AC, LM, CK, and SB wrote and approved the statistical analysis plan. AC, RM, LM, and CK did the formal analysis. CM, JT, and NW were responsible for study monitoring. AC wrote the original draft of the manuscript. AC, RM, PG, KG, DB, LM, ATLC, ND, SD, AD, TG, SRG, SH, CJ, RK, SaK, ShK, VK, DK, AL, AM, CM, MM, JO, MRG, SaS, VeKS, ViKS, JS, SuS, JT, PT, AMGV, NW, PK, GMV, CK, YL, and SB reviewed, edited, and approved the manuscript. AC, LM, CK, and SB verified the underlying data.

## ACKNOWLEDGEMENTS

We are grateful to the members of the External Advisory Panel, led by Prof. Nicholas J White (MORU), for their advice and guidance throughout the study. We also acknowledge the support of Kundavaram PP Abhilash (CMC), OC Abraham (CMC), T Balamugesh (CMC), Thambu David (CMC), Divya Dayanand (CMC), Biju George (CMC), Richa Gupta (CMC), Samuel G Hansdak (CMC), Ramya Iyadurai (CMC), Rajiv Karthik (CMC), Sharwar Kazmi (MSF), Mavuto Mukaka (MORU), Sowmya Sathyendra (CMC), Merylin Sebastian (CMC), Christopher Smith (LSHTM), Ramesh Vishwakarma (MORU), Sophie Yacoub (OUCRU), and Anand Zachariah (CMC).

## DATA SHARING

De-identified, individual participant data from this study will be available to researchers whose proposed purpose of use is approved by the data access committees at Médecins Sans Frontières and the Mahidol-Oxford Tropical Medicine Research Unit. Inquiries or requests for the data may be sent to data.sharing@london.msf.org and datasharing@tropmedres.ac. Researchers interested in accessing biobanked samples should contact the corresponding authors who will coordinate with the respective institutions.

## FUNDING

The PRIORITISE (Prognostication of Oxygen Requirement in Patients with Non-severe SARS-CoV-2 Infection) study was supported by MSF, India, who maintained a sponsor/investigator role for the study. The Wellcome Trust provides core funding to the Mahidol-Oxford Tropical Medicine Research Unit in Bangkok [220211, 215604/Z/19/Z], which supported the design, monitoring and analysis of the study. CK is supported by a Wellcome Trust/Royal Society Sir Henry Dale Fellowship [211182/Z/18/Z]. For the purpose of open access, the author has applied a CC BY public copyright license to any Author Accepted Manuscript version arising from this submission.

## DECLARATION OF INTERESTS

JO and SD report being employed by FIND the global alliance for diagnostic (https://www.finddx.org/) an organization dedicated to advancing the use of diagnostic tools. The other authors declare that they have no conflicts of interest.

## FIGURE LEGENDS

**Figure 1. Proportion of individuals fully vaccinated against COVID-19 as of 19 December 2021**. Adapted from https://ourworldindata.org/covid-vaccinations#country-by-country-data-on-vaccinations.[1]

**Figure 2. Screening and recruitment of participants into the PRIORITSE study.** *Reasons for exclusion: 64 vaccinated, 3 unable to provide consent, and 5 reason not documented. Towards the end of recruitment (March 2021 in AIIMS and May 2021 in CMC) vaccines against COVID-19 began to be rolled out in the study areas and a decision was made to exclude vaccinated participants as the study would not be powered to determine whether the prediction models were valid in this cohort.

**Figure 3. Performance measures and calibration plots for each model in the validation cohort**. Red line indicates perfect calibration; black dashed line indicates calibration slope for that particular model; blue rug plots indicate distribution of predicted risk for participants who did (top) and did not (bottom) meet the primary outcome. C-statistics indicate how well participants who met the primary outcome are differentiated from those who did not; perfect discrimination is indicated by a c-statistic of 1.0. Calibration slopes indicate agreement between predicted probabilities and observed outcomes; perfect calibration is indicated by a slope of 1.0.

**Figure 4. Decision curve analysis for each model in the validation cohort**. The net benefit for each model is compared to an “admit-all” (red line) and “admit-none” (green line) approach, and each model containing a biochemical biomarker (purple line) is also compared to the model containing only clinical variables (blue line). A threshold probability of 5% indicates a scenario where the value of 1 TP (patient admitted who will subsequently require oxygen) is equivalent to 19 FPs (patients admitted who will not subsequently require oxygen).

| Baseline characteristic | DEVELOPMENT COHORT | | | VALIDATION COHORT | | |
|---|---|---|---|---|---|---|
| | Overall (n = 257) | Developed oxygen requirement | | Overall (n = 166) | Developed oxygen requirement | |
| | | No (n = 207) | Yes (n = 50) | | No (n = 127) | Yes (n = 39) |
| **Demographics** | | | | | | |
| Age (years) | 52.0 (40.0 to 61.0) | 52.0 (40.0 to 60.0) | 54.0 (42.2 to 62.0) | 54.0 (41.2 to 63.0) | 55.0 (41.5 to 63.0) | 54.0 (41.0 to 66.0) |
| Male sex | 185 / 257 (72%) | 144 / 207 (70%) | 41 / 50 (82%) | 101 / 166 (61%) | 76 / 127 (60%) | 25 / 39 (64%) |
| BMI (kg/m$^2$) * | 26.0 (23.6 to 28.7) | 26.2 (23.8 to 28.8) | 25.8 (22.4 to 28.3) | 24.9 (23.4 to 27.6) | 24.8 (23.4 to 27.6) | 26.1 (23.7 to 27.6) |
| **Vital signs** | | | | | | |
| Heart rate (bpm) | 88.0 (80.0 to 97.0) | 86.0 (79.0 to 96.0) | 90.0 (86.0 to 99.5) | 84.0 (74.0 to 92.0) | 84.0 (74.0 to 90.0) | 84.0 (77.0 to 94.0) |
| Respiratory rate (bpm) | 22.0 (22.0 to 24.0) | 22.0 (22.0 to 24.0) | 22.0 (22.0 to 24.0) | 24.0 (22.0 to 24.0) | 22.0 (22.0 to 24.0) | 24.0 (22.0 to 24.0) |
| Oxygen saturation (%) | 98.0 (96.0 to 99.0) | 98.0 (97.0 to 99.0) | 96.0 (95.2 to 98.0) | 98.0 (96.0 to 99.0) | 98.0 (96.0 to 99.0) | 96.0 (95.5 to 98.0) |
| Axillary temperature (°C) | 36.8 (36.4 to 37.1) | 36.7 (36.4 to 37.0) | 36.9 (36.5 to 37.2) | 36.9 (36.7 to 37.2) | 36.9 (36.7 to 37.2) | 37.0 (36.9 to 37.2) |
| Systolic BP (mmHg) | 128.0 (116.0 to 138.0) | 128.0 (116.0 to 140.0) | 126.0 (118.0 to 134.8) | 121.0 (110.0 to 130.0) | 120.0 (110.0 to 130.0) | 122.0 (110.0 to 131.0) |
| Diastolic BP (mmHg) | 80.0 (72.0 to 88.0) | 80.0 (72.0 to 88.0) | 79.0 (70.0 to 88.0) | 76.0 (70.0 to 82.0) | 76.0 (70.0 to 82.0) | 74.0 (67.0 to 80.0) |
| qSOFA score ≥ 2 | 13 / 257 (5.1%) | 9 / 207 (4.3%) | 4 / 50 (8.0%) | 16 / 166 (9.6%) | 10 / 127 (7.9%) | 6 / 39 (15%) |
| **Comorbidities** | | | | | | |
| Current smokers | 10 / 257 (3.9%) | 8 / 207 (3.9%) | 2 / 50 (4.0%) | 4 / 166 (2.4%) | 3 / 127 (2.4%) | 1 / 39 (2.6%) |
| Reported comorbidity # | 165 / 257 (64%) | 128 / 207 (62%) | 37 / 50 (74%) | 117 / 166 (70%) | 91 / 127 (72%) | 26 / 39 (67%) |
| **Presenting illness** | | | | | | |
| Symptom duration (days) | 6.0 (4.0 to 8.0) | 6.0 (4.0 to 8.0) | 5.5 (5.0 to 7.0) | 6.0 (4.0 to 8.0) | 6.0 (3.5 to 8.0) | 5.0 (4.0 to 7.0) |
| History of fever | 243 / 257 (95%) | 196 / 207 (95%) | 47 / 50 (94%) | 155 / 166 (93%) | 118 / 127 (93%) | 37 / 39 (95%) |
| Breathlessness | 154 / 257 (60%) | 119 / 207 (57%) | 35 / 50 (70%) | 90 / 166 (54%) | 65 / 127 (51%) | 25 / 39 (64%) |
| Chest pain | 59 / 257 (23%) | 48 / 207 (23%) | 11 / 50 (22%) | 15 / 166 (9.0%) | 9 / 127 (7.1%) | 6 / 39 (15%) |

| | | | | | | |
|---|---|---|---|---|---|---|
| Abdominal pain | 35 / 257 (14%) | 32 / 207 (15%) | 3 / 50 (6.0%) | 15 / 166 (9.0%) | 12 / 127 (9.4%) | 3 / 39 (7.7%) |
| Diarrhoea | 80 / 257 (31%) | 65 / 207 (31%) | 15 / 50 (30%) | 47 / 166 (28%) | 33 / 127 (26%) | 14 / 39 (36%) |
| Severe myalgia | 140 / 257 (54%) | 110 / 207 (53%) | 30 / 50 (60%) | 75 / 166 (45%) | 65 / 127 (51%) | 10 / 39 (26%) |
| **Host biomarkers** | | | | | | |
| CRP (mg/l) * | 24.4 (3.9 to 88.9) | 17.9 (2.8 to 85.4) | 62.5 (19.7 to 134.4) | 58.1 (17.2 to 147.1) | 42.5 (12.3 to 111.9) | 95.8 (52.8 to 176.9) |
| D-dimer (ng/ml) * | 725.0 (382.4 to 1,466.4) | 640.6 (329.7 to 1,234.9) | 1,201.7 (679.9 to 2,307.0) | 968.2 (620.7 to 1,599.0) | 918.8 (579.0 to 1,454.9) | 1,148.1 (829.5 to 3,200.2) |
| IL-6 (pg/ml) * | 11.0 (4.9 to 36.2) | 8.7 (4.2 to 27.9) | 36.4 (18.4 to 70.7) | 31.6 (13.9 to 63.0) | 24.4 (11.4 to 47.2) | 71.1 (39.4 to 98.9) |
| NLR * | 3.2 (1.9 to 4.9) | 2.9 (1.7 to 4.5) | 4.4 (3.2 to 7.2) | 2.8 (1.8 to 5.4) | 2.5 (1.6 to 4.2) | 5.3 (2.7 to 7.0) |
| PCT (ng/ml) * | 0.1 (0.1 to 0.2) | 0.1 (0.1 to 0.1) | 0.1 (0.1 to 0.2) | 0.1 (0.1 to 0.2) | 0.1 (0.1 to 0.2) | 0.1 (0.1 to 0.3) |
| sTREM-1 (pg/ml) * | 378.0 (265.0 to 537.0) | 362.0 (259.0 to 522.0) | 424.5 (306.8 to 649.5) | 419.0 (285.0 to 596.8) | 389.0 (282.0 to 562.0) | 437.0 (349.0 to 660.8) |
| suPAR (ng/ml) | 4.2 (3.1 to 5.8) | 4.0 (2.9 to 5.5) | 5.4 (4.0 to 6.8) | 4.1 (3.1 to 5.6) | 3.8 (2.9 to 5.1) | 5.5 (3.9 to 6.7) |
| **Viral markers** | | | | | | |
| Ct value *§ | 26.0 (20.7 to 30.8) | 26.0 (20.6 to 30.1) | 26.4 (22.0 to 31.4) | 32.1 (28.3 to 36.2) | 32.8 (28.1 to 36.2) | 31.5 (28.4 to 36.0) |
| Seronegative *† | 117 / 252 (46%) | 90 / 203 (44%) | 27 / 49 (55%) | 73 / 160 (46%) | 51 / 123 (41%) | 22 / 37 (59%) |
| **Recruitment site** | | | | | | |
| CMC, Vellore | 133 / 257 (52%) | 110 / 207 (53%) | 23 / 50 (46%) | 166 / 166 (100%) | 127 / 127 (100%) | 39 / 39 (100%) |
| AIIMS, Patna | 124 / 257 (48%) | 97 / 207 (47%) | 27 / 50 (54%) | NA ‡ | NA ‡ | NA ‡ |

**Table 1. Baseline characteristics of development and validation cohorts, stratified by primary outcome status.** *Missing data: BMI = 1; CRP = 8, D-dimer = 3, IL-6 = 2, NLR = 12; PCT = 2; sTREM-1 = 2; Ct value = 181; serostatus = 11. #Details of the 12 comorbidities that participants were asked about are provided in Table S3. Comorbidities are not reliably diagnosed or known by patients in our contexts and therefore were not selected as one of the *a-priori* clinical predictors. [§]Different specimen collection procedures and PCR assays were used at each site (Table S9). [†]Seronegative defined as negative for both SARS-CoV-2 IgG and IgM antibodies. [‡]Recruitment closed in Patna in March 2021 and only participants from CMC were recruited into the temporal validation cohort. Median values (IQR) are reported for continuous variables. BMI = body mass index; BP = blood pressure; qSOFA = quick sequential organ failure assessment.

| Predicted probability of model (cut-off) | Per 100 patients (23 patients who would require oxygen) | | | | Ratio of incorrect to correct admissions (FP : TP) | Ratio of correct to incorrect discharges (TN : FN) |
|---|---|---|---|---|---|---|
| | Patients who would require oxygen admitted (TP) | Unnecessary hospital admissions (FP) | Patients who would require oxygen discharged (FN) | Patients correctly discharged (TN) | | |
| **Clinical model** | | | | | | |
| 0.1 | 21 | 58 | 2 | 19 | 3 to 1 | 10 to 1 |
| 0.15 | 18 | 46 | 5 | 31 | 3 to 1 | 6 to 1 |
| 0.20 | 14 | 29 | 9 | 48 | 2 to 1 | 5 to 1 |
| **IL-6 model** | | | | | | |
| 0.1 | 23 | 61 | 0 | 16 | 3 to 1 | NA |
| 0.15 | 21 | 49 | 2 | 28 | 2 to 1 | 14 to 1 |
| 0.20 | 19 | 38 | 4 | 39 | 2 to 1 | 10 to 1 |
| **NLR model** | | | | | | |
| 0.1 | 22 | 54 | 1 | 23 | 2 to 1 | 23 to 1 |
| 0.15 | 17 | 39 | 6 | 38 | 2 to 1 | 6 to 1 |
| 0.20 | 15 | 25 | 8 | 52 | 2 to 1 | 6 to 1 |
| **suPAR model** | | | | | | |
| 0.1 | 22 | 52 | 1 | 25 | 2 to 1 | 25 to 1 |
| 0.15 | 16 | 34 | 7 | 43 | 2 to 1 | 6 to 1 |
| 0.20 | 13 | 22 | 10 | 55 | 2 to 1 | 6 to 1 |
| **CRP model** | | | | | | |
| 0.1 | 21 | 54 | 2 | 23 | 3 to 1 | 12 to 1 |
| 0.15 | 20 | 43 | 3 | 34 | 2 to 1 | 11 to 1 |
| 0.20 | 16 | 36 | 7 | 41 | 2 to 1 | 6 to 1 |
| **D-dimer model** | | | | | | |
| 0.1 | 21 | 54 | 2 | 23 | 3 to 1 | 12 to 1 |
| 0.15 | 19 | 39 | 4 | 38 | 2 to 1 | 10 to 1 |
| 0.20 | 15 | 31 | 8 | 46 | 2 to 1 | 6 to 1 |
| **PCT model** | | | | | | |
| 0.1 | 21 | 57 | 2 | 20 | 3 to 1 | 10 to 1 |
| 0.15 | 18 | 45 | 5 | 32 | 2 to 1 | 6 to 1 |
| 0.20 | 14 | 27 | 9 | 50 | 2 to 1 | 6 to 1 |
| **sTREM-1 model** | | | | | | |
| 0.1 | 20 | 55 | 3 | 22 | 3 to 1 | 7 to 1 |
| 0.15 | 17 | 41 | 6 | 36 | 2 to 1 | 6 to 1 |
| 0.20 | 14 | 28 | 9 | 49 | 2 to 1 | 5 to 1 |

**Table 2. Predicted classification of patients at different cut-offs for each model, using the prevalence of the primary outcome in the validation cohort**. A cut-off of 0.1 reflects a management strategy in which any patient with a predicted risk of requiring oxygen ≥ 10% is admitted. FN = false negative; FP = false positive; TN = true negative; TP = true positive.

Figure 1

Accepted

Figure 2

Accepte

Figure 3

Figure 4

Clinical model
Clinical and CRP models
Clinical and D-Dimer models
Clinical and IL-6 models
Clinical and NLR models
Clinical and PCT models
Clinical and sTREM-1 models
Clinical and suPAR models
Net Benefit
0.20
0.15
0.10
0.05
0.00
0%
10%
20%
30%
40%
Threshold probability

## Supplementary Material

ciac224_suppl_Supplementary_Appendix

