# Facilitating Safe Discharge Through Predicting Disease Progression in Moderate Coronavirus Disease 2019 (COVID-19): A Prospective Cohort Study to Develop and Validate a Clinical Prediction Model in Resource-Limited Settings

**DOI:** 10.1093/cid/ciac224

**Published:** 2022-03-21

**Authors:** Arjun Chandna, Raman Mahajan, Priyanka Gautam, Lazaro Mwandigha, Karthik Gunasekaran, Divendu Bhusan, Arthur T L Cheung, Nicholas Day, Sabine Dittrich, Arjen Dondorp, Tulasi Geevar, Srinivasa R Ghattamaneni, Samreen Hussain, Carolina Jimenez, Rohini Karthikeyan, Sanjeev Kumar, Shiril Kumar, Vikash Kumar, Debasree Kundu, Ankita Lakshmanan, Abi Manesh, Chonticha Menggred, Mahesh Moorthy, Jennifer Osborn, Melissa Richard-Greenblatt, Sadhana Sharma, Veena K Singh, Vikash K Singh, Javvad Suri, Shuichi Suzuki, Jaruwan Tubprasert, Paul Turner, Annavi M G Villanueva, Naomi Waithira, Pragya Kumar, George M Varghese, Constantinos Koshiaris, Yoel Lubell, Sakib Burza

**Affiliations:** Cambodia Oxford Medical Research Unit, Angkor Hospital for Children, Siem Reap, Cambodia; Centre for Tropical Medicine & Global Health, University of Oxford, Oxford, United Kingdom; Médecins Sans Frontières, New Delhi, India; Department of Infectious Diseases, Christian Medical College, Vellore, India; Nuffield Department of Primary Care Health Sciences, University of Oxford, Oxford, United Kingdom; Department of Medicine, Christian Medical College, Vellore, India; Department of Internal Medicine, All India Institute of Medical Sciences, Patna, India; Centre for Tropical Medicine & Global Health, University of Oxford, Oxford, United Kingdom; Mahidol Oxford Tropical Medicine Research Unit, Mahidol University, Bangkok, Thailand; Centre for Tropical Medicine & Global Health, University of Oxford, Oxford, United Kingdom; Mahidol Oxford Tropical Medicine Research Unit, Mahidol University, Bangkok, Thailand; Centre for Tropical Medicine & Global Health, University of Oxford, Oxford, United Kingdom; Foundation for Innovative Diagnostics, Geneva, Switzerland; Centre for Tropical Medicine & Global Health, University of Oxford, Oxford, United Kingdom; Mahidol Oxford Tropical Medicine Research Unit, Mahidol University, Bangkok, Thailand; Department of Transfusion Medicine & Immunohaematology, Christian Medical College, Vellore, India; Médecins Sans Frontières, New Delhi, India; Médecins Sans Frontières, New Delhi, India; Médecins Sans Frontières, New Delhi, India; Department of Infectious Diseases, Christian Medical College, Vellore, India; Department of Cardiothoracic & Vascular Surgery, All India Institute of Medical Sciences, Patna, India; Department of Virology, Rajendra Memorial Research Institute of Medical Sciences, Patna, India; Médecins Sans Frontières, New Delhi, India; Department of Infectious Diseases, Christian Medical College, Vellore, India; Médecins Sans Frontières, New Delhi, India; Department of Infectious Diseases, Christian Medical College, Vellore, India; Mahidol Oxford Tropical Medicine Research Unit, Mahidol University, Bangkok, Thailand; Department of Clinical Virology, Christian Medical College, Vellore, India; Foundation for Innovative Diagnostics, Geneva, Switzerland; Perelman School of Medicine, University of Pennsylvania, Philadelphia, Pennsylvania, USA; Department of Biochemistry, All India Institute of Medical Sciences, Patna, India; Department of Burns & Plastic Surgery, All India Institute of Medical Sciences, Patna, India; Médecins Sans Frontières, New Delhi, India; Médecins Sans Frontières, New Delhi, India; School of Tropical Medicine & Global Health, Nagasaki University, Nagasaki, Japan; Mahidol Oxford Tropical Medicine Research Unit, Mahidol University, Bangkok, Thailand; Cambodia Oxford Medical Research Unit, Angkor Hospital for Children, Siem Reap, Cambodia; Centre for Tropical Medicine & Global Health, University of Oxford, Oxford, United Kingdom; School of Tropical Medicine & Global Health, Nagasaki University, Nagasaki, Japan; Centre for Tropical Medicine & Global Health, University of Oxford, Oxford, United Kingdom; Mahidol Oxford Tropical Medicine Research Unit, Mahidol University, Bangkok, Thailand; Department of Community & Family Medicine, All India Institute of Medical Sciences, Patna, Indiaand; Department of Infectious Diseases, Christian Medical College, Vellore, India; Nuffield Department of Primary Care Health Sciences, University of Oxford, Oxford, United Kingdom; Centre for Tropical Medicine & Global Health, University of Oxford, Oxford, United Kingdom; Mahidol Oxford Tropical Medicine Research Unit, Mahidol University, Bangkok, Thailand; Médecins Sans Frontières, New Delhi, India; Department of Clinical Research, London School of Hygiene & Tropical Medicine, London, United Kingdom

**Keywords:** COVID-19, prognostic model, triage, low- and middle-income country, LMIC

## Abstract

**Background:**

In locations where few people have received coronavirus disease 2019 (COVID-19) vaccines, health systems remain vulnerable to surges in severe acute respiratory syndrome coronavirus 2 (SARS-CoV-2) infections. Tools to identify patients suitable for community-based management are urgently needed.

**Methods:**

We prospectively recruited adults presenting to 2 hospitals in India with moderate symptoms of laboratory-confirmed COVID-19 to develop and validate a clinical prediction model to rule out progression to supplemental oxygen requirement. The primary outcome was defined as any of the following: SpO_2 _< 94%; respiratory rate > 30 BPM; SpO_2_/FiO_2 _< 400; or death. We specified a priori that each model would contain three clinical parameters (age, sex, and SpO_2_) and 1 of 7 shortlisted biochemical biomarkers measurable using commercially available rapid tests (C-reactive protein [CRP], D-dimer, interleukin 6 [IL-6], neutrophil-to-lymphocyte ratio [NLR], procalcitonin [PCT], soluble triggering receptor expressed on myeloid cell-1 [sTREM-1], or soluble urokinase plasminogen activator receptor [suPAR]), to ensure the models would be suitable for resource-limited settings. We evaluated discrimination, calibration, and clinical utility of the models in a held-out temporal external validation cohort.

**Results:**

In total, 426 participants were recruited, of whom 89 (21.0%) met the primary outcome; 257 participants comprised the development cohort, and 166 comprised the validation cohort. The 3 models containing NLR, suPAR, or IL-6 demonstrated promising discrimination (c-statistics: 0.72–0.74) and calibration (calibration slopes: 1.01–1.05) in the validation cohort and provided greater utility than a model containing the clinical parameters alone.

**Conclusions:**

We present 3 clinical prediction models that could help clinicians identify patients with moderate COVID-19 suitable for community-based management. The models are readily implementable and of particular relevance for locations with limited resources.

## Teaser

We report 3 clinical prediction models to help identify which patients with moderate COVID-19 can be safely managed in the community. Each model contains 3 easily ascertained clinical parameters and 1 biochemical biomarker, measurable with a commercially available rapid test.

**Figure 1.**
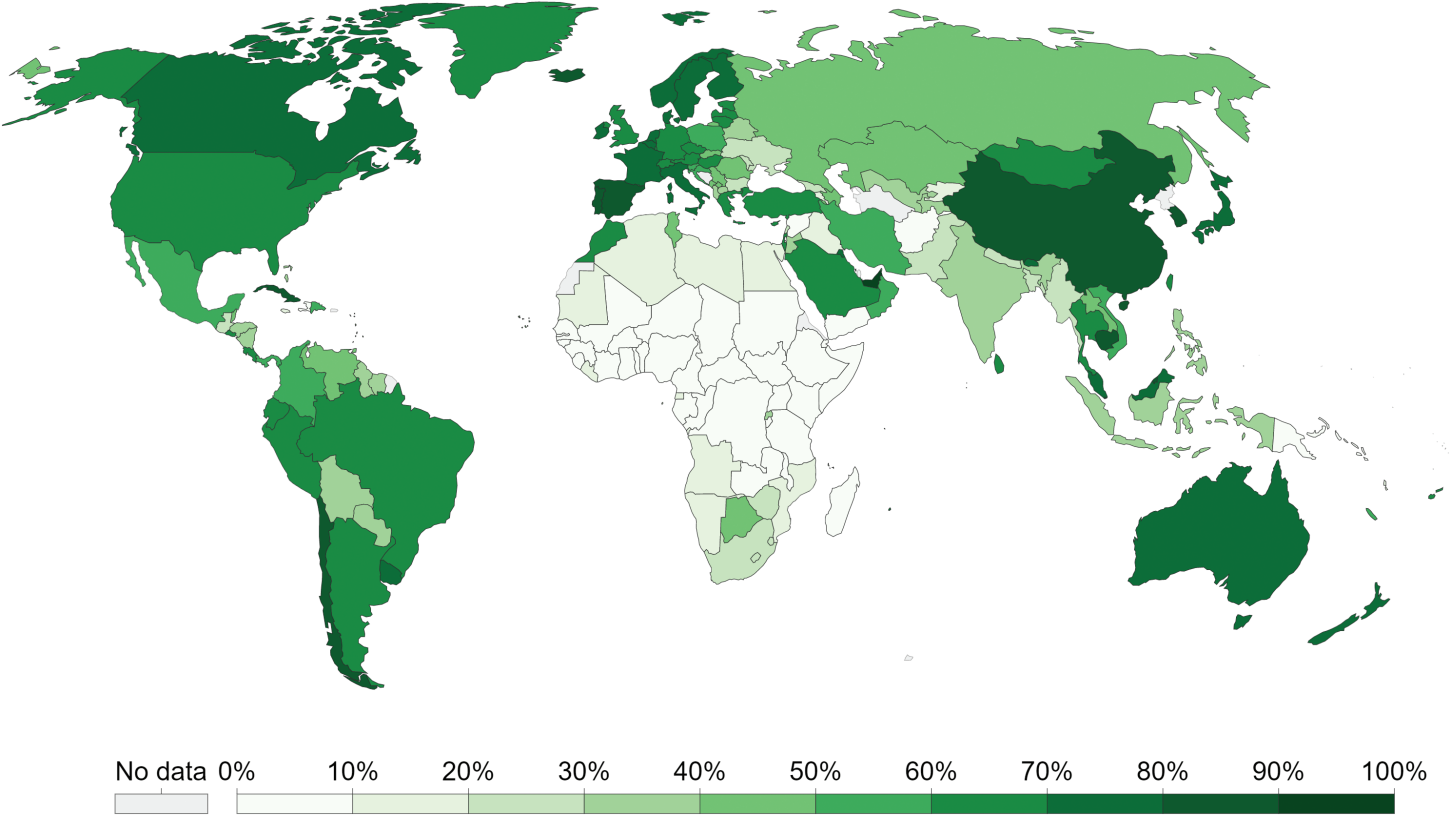
Proportion of individuals fully vaccinated against COVID-19 as of 19 December 2021. Adapted from https://ourworldindata.org/covid-vaccinations#country-by-country-data-on-vaccinations [1]. Abbreviation: COVID-19, coronavirus disease 2019.

In low-income countries, where fewer than 5% of people have received a coronavirus disease 2019 (COVID-19) vaccine [1] fragile healthcare systems remain vulnerable to being overwhelmed by a surge in COVID-19 cases (Figure 1) [2–4].

A minority of patients with COVID-19 require admission to hospital. Oxygen is the most important supportive treatment and in most low- and middle-income countries (LMICs) is the practical ceiling of care [5]. The World Health Organization (WHO) estimates that 15% of patients with symptomatic COVID-19 will require supplemental oxygen [6]. Effective identification of patients who are unlikely to become hypoxic would have considerable benefit; tools to support triage could decompress healthcare systems by giving practitioners confidence to allocate resources more efficiently [7].

Numerous prognostic models for COVID-19 have been developed [8, 9]. Almost all predict critical illness or mortality and thus cannot inform whether a patient might be safely managed in the community. Of those that focus on patients with moderate disease, most rely on retrospective or registry-based data [10–14], lack external validation [15, 16], and are not feasible for use in resource-limited settings [9, 17]. Moreover, most existing studies did not follow best-practice guidelines for model building and reporting [18], are at high risk of bias [8], and the resulting models are neither suitable nor recommended for use in LMIC contexts [9].

We set out to develop and validate a clinical prediction model to rule out progression to supplemental oxygen requirement in patients presenting with moderate COVID-19. We hypothesized that combining simple clinical parameters with host biomarkers feasible for measurement in resource-limited settings and implicated in the pathogenesis of COVID-19 would improve prognostication.

## METHODS

### Study Population

PRIORITISE is a prospective observational cohort study. Consecutive patients aged ≥ 18 years with clinically suspected severe acute respiratory syndrome coronavirus 2 (SARS-CoV-2) infection presenting with moderate symptoms to the All India Institute of Medical Sciences (AIIMS) Hospital in Patna, India, and the Christian Medical College (CMC) Hospital in Vellore, India, were screened (daytime hours, Monday to Saturday). AIIMS is a 1000-bed hospital and the largest medical facility providing primary-to-tertiary healthcare in the state of Bihar. CMC is a 3000-bed not-for-profit hospital that provided care for ~1500 patients with COVID-19 each day during the peak of the Delta-wave surge in India.

We adapted the case definitions in the World Health Organization (WHO) Clinical Management guideline (moderate disease) [6] and WHO Clinical Progression Scale (WHO-CPS; scores 2, 3, or 4) [19] to define moderate disease as follows: a peripheral oxygen saturation (SpO_2_) ≥ 94% and respiratory rate < 30 breaths per minute (BPM), in the context of systemic symptoms (breathlessness or fever and chest pain, abdominal pain, diarrhea, or severe myalgia), recognizing that the threshold for hospitalization varies throughout a pandemic and that a sensitive cutoff for hypoxia would be desirable in a tool to inform community-based management [19, 20].

### Data Collection

Structured case-report forms (Supplementary Materials 2–10) were completed at enrolment, day 7, and day 14, and daily during admission to the study facilities. Anthropometrics and vital signs were measured at enrolment and demographics, clinical symptoms, comorbidities, and medication history collected via brief interview with the participant. Venous blood samples were collected at enrollment in ethylenediaminetetraacetic acid (EDTA) tubes. Participants were followed-up in-person when admitted to the facility and by telephone on days 7 and 14 if discharged prior to this. Those discharged who reported worsening symptoms on day 7 and/or persistent symptoms on day 14 were recalled to have their SpO_2_ and respiratory rate measured.

### Primary Outcome

The primary outcome was development of an oxygen requirement within 14 days of enrollment, defined as any of the following: SpO_2 _< 94%; respiratory rate > 30 BPM; SpO_2_/FiO_2 _< 400 [21, 22]; or death, aligning closely with a WHO-CPS score of ≥ 5 [19]. Patients who received supplemental oxygen outside the study facilities were classified as meeting the primary outcome if it was not possible to retrieve their case notes, provided that the oxygen was prescribed in a licensed medical facility. The site study teams were unaware of which baseline variables had been preselected as candidate predictors when determining outcome status.

### Candidate Predictors

We decided a priori that a model using 4 predictors would be practical in high-patient-throughput resource-limited settings. Considering resource constraints, reliability, validity, feasibility, and biological plausibility, we prespecified that each model would contain age, sex, SpO_2_, and 1 biochemical biomarker [10, 17, 23].

Following a literature review (Supplementary Figures 1–2), biomarkers were shortlisted in consultation with FIND, the global alliance for diagnostics (Geneva, Switzerland). To qualify for inclusion, biomarkers had to be quantifiable with rapid tests in clinical use or late-stage development (Technology Readiness Level ≥ 4; Supplementary Table 1) [24]. The final list included: C-reactive protein (CRP), D-dimer, interleukin 6 (IL-6), neutrophil-to-lymphocyte ratio (NLR), procalcitonin (PCT), soluble triggering receptor expressed on myeloid cells-1 (sTREM-1), and soluble urokinase plasminogen activator receptor (suPAR) [25–29].

Clinical predictors were measured at enrolment and all biomarkers except NLR were measured retrospectively from samples obtained at enrollment. NLR was measured on site and was not repeated if it had been measured at the site within 24 hours prior to recruitment. All predictors were measured blinded to outcome status.

### Laboratory Procedures

Complete blood counts (XP-300-Hematology-Analyzer, Sysmex, Lincolnshire, Illinois, USA) were performed on site, and aliquots of EDTA-plasma were stored at −20°C or below until testing. Biomarker concentrations were quantified using the suPARnostic ELISA (ViroGates, Denmark) and Simple Plex Ella microfluidic platform (ProteinSimple, San Jose, California, USA) as described elsewhere [30]. Remaining plasma was biobanked on site. SARS-CoV-2 immunoglobulin G (IgG) and immunoglobulin M (IgM) antibodies were measured using the SCoV-2 Detect ELISA (InBios, Seattle, Washington, USA). Oral and/or nasopharyngeal swabs were collected to confirm SARS-CoV-2 infection via reverse transcription polymerase chain reaction (RT-PCR) (Cepheid Xpert Xpress SARS-CoV-2, Sunnyvale, California or Altona RealStar SARS-CoV-2 rRT-PCR, Germany).

### Sample Size

We considered the sample size for model development and validation separately. We followed the recommendations of Riley et al and assumed a conservative *R*^2^ Nagelkerke of 0.15 [31]. We anticipated that ~8% of participants would meet the primary endpoint and estimated that 44 outcome events would be required to derive a prediction model comprising four candidate predictors and minimize the risk of overfitting (events per parameter [EPP] = 11).

Given the uncertainty around deterioration rates amongst patients with moderate COVID-19 at the time of study inception, we prespecified an interim review after the first 100 participants were recruited. At this review, the proportion of participants meeting the primary endpoint was higher than anticipated (20% vs. 8%). At this higher prevalence, and using *R*^2^ values from 0.20 to 0.15, between 52 and 68 outcome events (EPP = 13–17) would be required to develop the prediction models [31]. Recognizing that (i) our range of *R*^2^ estimates was conservative, (ii) penalized regression methods would reduce the risk of overfitting, and (iii) the external validation cohort would allow assessment of model optimism, and following the advice of the External Advisory Panel, a decision was made to use the first 50 outcome events to derive the models. Participants recruited after that point were entered into the external temporal validation cohort.

### Model Development and Validation

We explored the relationship between candidate predictors and the primary outcome using a Lowess smoothing approach to identify nonlinear patterns. Transformations were used when serious violations of linearity were detected. We used penalized logistic (ridge) regression to develop the models and shrink regression coefficients to minimize model optimism. All predictors were prespecified, and no predictor selection was performed during model development. Due to few missing data (< 3% for any single predictor), missing observations were replaced with the median value, grouped by outcome status. A sensitivity analysis was conducted using full-case analysis.

We assessed discrimination (c-statistics) and calibration (calibration plots and slopes) for each model in the validation cohort, and examined classifications (true positives [TP], false positives [FP], true negatives [TN], false negatives [FN]) at clinically relevant cut-points (predicted probabilities). Finally, recognizing that the relative value of a TP and FP will vary at different stages of the pandemic [20], we examined the potential clinical utility of the models using decision curve analyses to quantify the net benefit between correctly identified TP or TN and incorrectly identified FP or FN at a range of plausible trade-offs (threshold probabilities) [32].

All analyses were done in R v4.03.

### Ethical Approvals

This investigator-initiated study was prospectively registered (ClinicalTrials.gov; NCT04441372), with protocol and statistical analysis plan uploaded to the Open Science Framework platform (DOI: 10.17605/OSF.IO/DXQ43). Ethical approval was given by the AIIMS, Patna Ethics Committee; CMC Ethics Committee; Oxford Tropical Research Ethics Committee; and MSF Ethical Review Board.

## RESULTS

**Figure 2.**
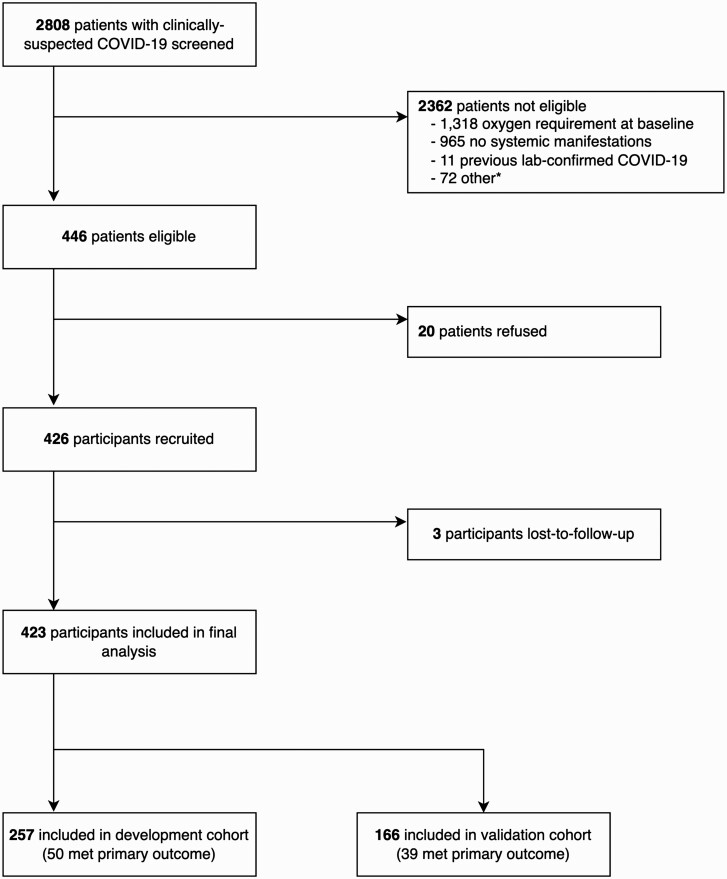
Screening and recruitment of participants into the PRIORITSE study. ^*^Reasons for exclusion: 64 vaccinated, 3 unable to provide consent, and 5 reason not documented. Toward the end of recruitment (March 2021 in AIIMS and May 2021 in CMC) vaccines against COVID-19 began to be rolled out in the study areas and a decision was made to exclude vaccinated participants as the study would not be powered to determine whether the prediction models were valid in this cohort. Abbreviations: AIIMS, All India Institute of Medical Sciences; CMC, Christian Medical College; COVID-19, coronavirus disease 2019.

Between 22 October 2020 and 3 July 2021, 2808 patients with clinically suspected COVID-19 were screened, of whom 446 were eligible (446/2808; 15.9%) and 426 were recruited (20/446; 4.5% refusal rate). Three participants were lost to follow-up (3/426; 0.7%) and excluded from further analyses (Figure 2). All participants had laboratory-confirmed SARS-CoV-2 infection (421/423 [99.5%] via RT-PCR). The maximum amount of missing data for any predictor was 2.6% (NLR; 11/423; Supplementary Table 2). The first 257 participants comprised the development cohort, and the remaining 166 participants comprised the temporal validation cohort.

### Outcomes

Development and validation cohorts were largely balanced with respect to baseline characteristics (Table 1; Supplementary Table 3). There was a higher proportion of males in the development cohort (72% [185/257] vs. 61% [101/166]). In the validation cohort, more participants had a qSOFA score ≥ 2 (16/166 [9.6%] vs. 13/257 [5.1%]), and the validation cohort had higher median CRP (58.1 mg/L vs. 24.4 mg/L) and IL-6 (31.6 pg/mL vs. 11.0 pg/mL) concentrations. Eighty-nine participants met the primary outcome (89/423; 21.0%); 50 in the development cohort (50/257; 19.5%) and 39 in the validation cohort (39/166; 23.5%). Median (interquartile range [IQR]) time to oxygen requirement was 1 (1–3) day; 11 participants died, 2 were mechanically ventilated, 15 received noninvasive ventilation, 49 received oxygen via a face mask and/or nasal cannula (1 outside the study facilities), and 12 had an SpO_2_ < 94% but did not receive oxygen supplementation (Supplementary Table 4; Supplementary Figure 3).

**Table 1.** Baseline Characteristics of Development and Validation Cohorts, Stratified by Primary Outcome Status

Baseline Characteristic	Development Cohort			Validation Cohort		
	Overall (n = 257)	Developed Oxygen Requirement		Overall (n = 166)	Developed Oxygen Requirement	
		No (n = 207)	Yes (n = 50)		No (n = 127)	Yes (n = 39)
**Demographics**						
Age (years)	52.0 (40.0–61.0)	52.0 (40.0–60.0)	54.0 (42.2–62.0)	54.0 (41.2–63.0)	55.0 (41.5–63.0)	54.0 (41.0–66.0)
Male sex	185/257 (72%)	144/207 (70%)	41/50 (82%)	101/166 (61%)	76/127 (60%)	25/39 (64%)
BMI (kg/m²)^a^	26.0 (23.6–28.7)	26.2 (23.8–28.8)	25.8 (22.4–28.3)	24.9 (23.4–27.6)	24.8 (23.4–27.6)	26.1 (23.7–27.6)
**Vital signs**						
Heart rate (BPM)	88.0 (80.0–97.0)	86.0 (79.0–96.0)	90.0 (86.0–99.5)	84.0 (74.0–92.0)	84.0 (74.0–90.0)	84.0 (77.0–94.0)
Respiratory rate (BPM)	22.0 (22.0–24.0)	22.0 (22.0–24.0)	22.0 (22.0 to 24.0)	24.0 (22.0 to 24.0)	22.0 (22.0–24.0)	24.0 (22.0–24.0)
Oxygen saturation (%)	98.0 (96.0–99.0)	98.0 (97.0–99.0)	96.0 (95.2–98.0)	98.0 (96.0–99.0)	98.0 (96.0–99.0)	96.0 (95.5–98.0)
Axillary temperature (°C)	36.8 (36.4–37.1)	36.7 (36.4–37.0)	36.9 (36.5–37.2)	36.9 (36.7–37.2)	36.9 (36.7–37.2)	37.0 (36.9–37.2)
Systolic BP (mmHg)	128.0 (116.0–138.0)	128.0 (116.0–140.0)	126.0 (118.0–134.8)	121.0 (110.0–130.0)	120.0 (110.0–130.0)	122.0 (110.0–131.0)
Diastolic BP (mmHg)	80.0 (72.0–88.0)	80.0 (72.0–88.0)	79.0 (70.0–88.0)	76.0 (70.0–82.0)	76.0 (70.0–82.0)	74.0 (67.0–80.0)
qSOFA score ≥ 2	13/257 (5.1%)	9/207 (4.3%)	4/50 (8.0%)	16/166 (9.6%)	10/127 (7.9%)	6/39 (15%)
**Comorbidities**						
Current smokers	10/257 (3.9%)	8/207 (3.9%)	2/50 (4.0%)	4/166 (2.4%)	3/127 (2.4%)	1/39 (2.6%)
Reported comorbidity^b^	165/257 (64%)	128/207 (62%)	37/50 (74%)	117/166 (70%)	91/127 (72%)	26/39 (67%)
**Presenting illness**						
Symptom duration (days)	6.0 (4.0–8.0)	6.0 (4.0–8.0)	5.5 (5.0–7.0)	6.0 (4.0–8.0)	6.0 (3.5–8.0)	5.0 (4.0–7.0)
History of fever	243/257 (95%)	196/207 (95%)	47/50 (94%)	155/166 (93%)	118/127 (93%)	37/39 (95%)
Breathlessness	154/257 (60%)	119/207 (57%)	35/50 (70%)	90/166 (54%)	65/127 (51%)	25/39 (64%)
Chest pain	59/257 (23%)	48/207 (23%)	11/50 (22%)	15/166 (9.0%)	9/127 (7.1%)	6/39 (15%)
Abdominal pain	35/257 (14%)	32/207 (15%)	3/50 (6.0%)	15/166 (9.0%)	12/127 (9.4%)	3/39 (7.7%)
Diarrhea	80/257 (31%)	65/207 (31%)	15/50 (30%)	47/166 (28%)	33/127 (26%)	14/39 (36%)
Severe myalgia	140/257 (54%)	110/207 (53%)	30/50 (60%)	75/166 (45%)	65/127 (51%)	10/39 (26%)
**Host biomarkers**						
CRP (mg/L)^a^	24.4 (3.9–88.9)	17.9 (2.8–85.4)	62.5 (19.7–134.4)	58.1 (17.2–147.1)	42.5 (12.3–111.9)	95.8 (52.8–176.9)
D-dimer (ng/mL)^a^	725.0 (382.4–1,466.4)	640.6 (329.7–1,234.9)	1,201.7 (679.9–2,307.0)	968.2 (620.7–1,599.0)	918.8 (579.0–1,454.9)	1,148.1 (829.5–3,200.2)
IL-6 (pg/mL)^a^	11.0 (4.9–36.2)	8.7 (4.2–27.9)	36.4 (18.4–70.7)	31.6 (13.9–63.0)	24.4 (11.4–47.2)	71.1 (39.4–98.9)
NLR^a^	3.2 (1.9–4.9)	2.9 (1.7–4.5)	4.4 (3.2–7.2)	2.8 (1.8–5.4)	2.5 (1.6–4.2)	5.3 (2.7–7.0)
PCT (ng/mL)^a^	0.1 (0.1–0.2)	0.1 (0.1–0.1)	0.1 (0.1–0.2)	0.1 (0.1–0.2)	0.1 (0.1–0.2)	0.1 (0.1–0.3)
sTREM-1 (pg/mL)^a^	378.0 (265.0–537.0)	362.0 (259.0–522.0)	424.5 (306.8–649.5)	419.0 (285.0–596.8)	389.0 (282.0–562.0)	437.0 (349.0–660.8)
suPAR (ng/mL)	4.2 (3.1–5.8)	4.0 (2.9–5.5)	5.4 (4.0–6.8)	4.1 (3.1–5.6)	3.8 (2.9–5.1)	5.5 (3.9–6.7)
**Viral markers**						
Ct value^a,^^c^	26.0 (20.7–30.8)	26.0 (20.6–30.1)	26.4 (22.0–31.4)	32.1 (28.3–36.2)	32.8 (28.1–36.2)	31.5 (28.4–36.0)
Seronegative^a,^^d^	117/252 (46%)	90/203 (44%)	27/49 (55%)	73/160 (46%)	51/123 (41%)	22/37 (59%)
**Recruitment site**						
CMC, Vellore	133/257 (52%)	110/207 (53%)	23/50 (46%)	166/166 (100%)	127/127 (100%)	39/39 (100%)
AIIMS, Patna	124/257 (48%)	97/207 (47%)	27/50 (54%)	NA^e^	NA^e^	NA^e^

Median values IQR are reported for continuous variables.

Abbreviations: AIIMS, All India Institute of Medical Sciences; BMI, body mass index; BP, blood pressure; BPM, beats per minute; CMC, Christian Medical College; CRP, C-reactive protein; IgG, immunoglobulin G; IgM, immunoglobulin M; IL-6, interleukin 6; IQR, interquartile range; NA, not applicable; NLR, neutrophil-to-lymphocyte ratio; PCR, polymerase chain reaction; PCT, procalcitonin; qSOFA, quick sequential organ failure assessment; SARS-CoV-2, severe acute respiratory syndrome coronavirus 2; sTREM-1, soluble triggering receptor expressed on myeloid cell-1; suPAR, soluble urokinase plasminogen activator receptor.

Missing data: BMI = 1; CRP = 8, D-dimer = 3, IL-6 = 2, NLR = 12; PCT = 2; sTREM-1 = 2; Ct value = 181; serostatus = 11.

Details of the 12 comorbidities that participants were asked about are provided in Supplementary Table 3. Comorbidities are not reliably diagnosed or known by patients in our contexts and therefore were not selected as 1 of the a priori clinical predictors.

Different specimen collection procedures and PCR assays were used at each site (Supplementary Table 9).

Seronegative defined as negative for both SARS-CoV-2 IgG and IgM antibodies.

Recruitment closed in AIIMS in March 2021, and only participants from CMC were recruited into the temporal validation cohort.

Relationships between candidate predictors and the primary outcome are illustrated (Supplementary Figure 4), and c-statistics (continuous predictors) and odds ratios (continuous and categorical predictors) reported (Supplementary Table 5). The full models are presented in the Supplementary Materials (Supplementary Table 5; Supplementary Figure 5). After adjustment for the 3 clinical variables, 5 biomarkers (CRP, D-dimer, IL-6, NLR, and suPAR) were independently associated with development of an oxygen requirement.

### Prognostic Models

**Figure 3.**
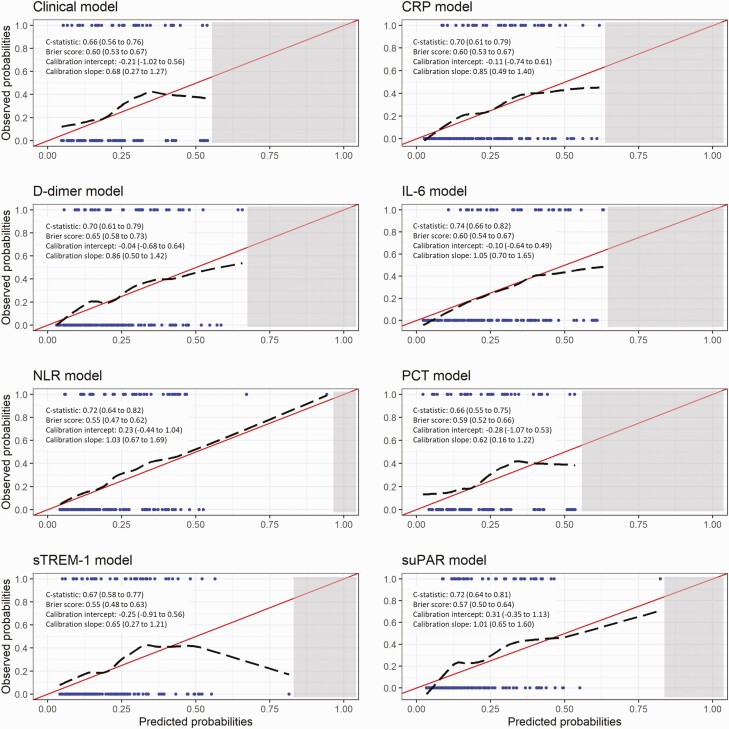
Performance measures and calibration plots for each model in the validation cohort. Red line indicates perfect calibration; black dashed line indicates calibration slope for that particular model; blue rug plots indicate distribution of predicted risk for participants who did (top) and did not (bottom) meet the primary outcome; grey shaded rectangle indicates region within which no individual participant’s predicted risk falls for that particular model. C-statistics indicate how well participants who met the primary outcome are differentiated from those who did not; perfect discrimination is indicated by a c-statistic of 1.0. Calibration slopes indicate agreement between predicted probabilities and observed outcomes; perfect calibration is indicated by a slope of 1.0. Abbreviations: CRP, C-reactive protein; IL-6, interleukin 6; NLR, neutrophil-to-lymphocyte ratio; PCT, procalcitonin; sTREM-1, soluble triggering receptor expressed on myeloid cell-1; suPAR, soluble urokinase plasminogen activator receptor.

Discrimination and calibration of each model in the validation cohort are presented in Figure 3. C-statistics ranged from 0.66 (clinical model and model containing PCT) to 0.74 (model containing IL-6). Calibration slopes ranged from 0.62 (model containing PCT) to 1.01 (model containing suPAR). Calibration was better at lower predicted probabilities, with some models overestimating risk at higher predicted probabilities.

The ability of each model to rule out progression to oxygen requirement amongst patients with moderate COVID-19 at predicted probabilities (cutoffs) of 10%, 15%, and 20% is shown (Table 2; Supplementary Table 6; Supplementary Figure 6). A cutoff of 10% reflects a management strategy equivalent to admitting any patient in whom the predicted risk of developing an oxygen requirement is ≥ 10%. At this cutoff, the results suggest that a model containing the three clinical parameters (age, sex, and SpO_2_) without any biomarkers could facilitate correctly sending home ~25% of patients with moderate COVID-19 who would not subsequently require supplemental oxygen, at the cost of also sending home ~9% of moderate patients who would deteriorate and require supplemental oxygen, that is, a ratio of correctly to incorrectly discharged patients of 10:1.

**Table 2.** Predicted Classification of Patients at Different Cutoffs for Each Model, Using the Prevalence of the Primary Outcome in the Validation Cohort

**Predicted Probability of Model (Cutoff)**	**Per 100 Patients (23 Patients Who Would Require Oxygen)**				**Ratio of Incorrect to Correct Admissions (FP: TP)**	**Ratio of Correct to Incorrect Discharges (TN: FN)**
	**Patients Who Would Require Oxygen Admitted (TP)**	**Unnecessary Hospital Admissions (FP)**	**Patients Who Would Require Oxygen Discharged (FN)**	**Patients Correctly Discharged (TN)**		
**Clinical model**						
0.1	21	58	2	19	3 to 1	10 to 1
0.15	18	46	5	31	3 to 1	6 to 1
0.20	14	29	9	48	2 to 1	5 to 1
**IL-6 model**						
0.1	23	61	0	16	3 to 1	NA
0.15	21	49	2	28	2 to 1	14 to 1
0.20	19	38	4	39	2 to 1	10 to 1
**NLR model**						
0.1	22	54	1	23	2 to 1	23 to 1
0.15	17	39	6	38	2 to 1	6 to 1
0.20	15	25	8	52	2 to 1	6 to 1
**suPAR model**						
0.1	22	52	1	25	2 to 1	25 to 1
0.15	16	34	7	43	2 to 1	6 to 1
0.20	13	22	10	55	2 to 1	6 to 1
**CRP model**						
0.1	21	54	2	23	3 to 1	12 to 1
0.15	20	43	3	34	2 to 1	11 to 1
0.20	16	36	7	41	2 to 1	6 to 1
**D-dimer model**						
0.1	21	54	2	23	3 to 1	12 to 1
0.15	19	39	4	38	2 to 1	10 to 1
0.20	15	31	8	46	2 to 1	6 to 1
**PCT model**						
0.1	21	57	2	20	3 to 1	10 to 1
0.15	18	45	5	32	2 to 1	6 to 1
0.20	14	27	9	50	2 to 1	6 to 1
**sTREM-1 model**						
0.1	20	55	3	22	3 to 1	7 to 1
0.15	17	41	6	36	2 to 1	6 to 1
0.20	14	28	9	49	2 to 1	5 to 1

A cutoff of 0.1 reflects a management strategy in which any patient with a predicted risk of requiring oxygen ≥ 10% is admitted.

Abbreviations: CRP, C-reactive protein; FN, false negative; FP, false positive; IL-6, interleukin 6; NLR, neutrophil-to-lymphocyte ratio; PCT, procalcitonin; sTREM-1, soluble triggering receptor expressed on myeloid cell-1; suPAR, soluble urokinase plasminogen activator receptor; TN, true negative; TP, true positive.

The inclusion of either NLR or suPAR improved the predictive performance such that the ratio of correctly to incorrectly discharged patients increased to 23:1 or 25:1 respectively, whilst a model containing IL-6 resulted in a similar proportion (~21%) of correctly discharged patients as the clinical model but without missing any patients who would deteriorate and require supplemental oxygen. Inclusion of the other candidate biomarkers (CRP, D-dimer, PCT, or sTREM-1) did not improve the ability of the clinical model to rule out progression to supplemental oxygen requirement.

### Generalizability

**Figure 4.**
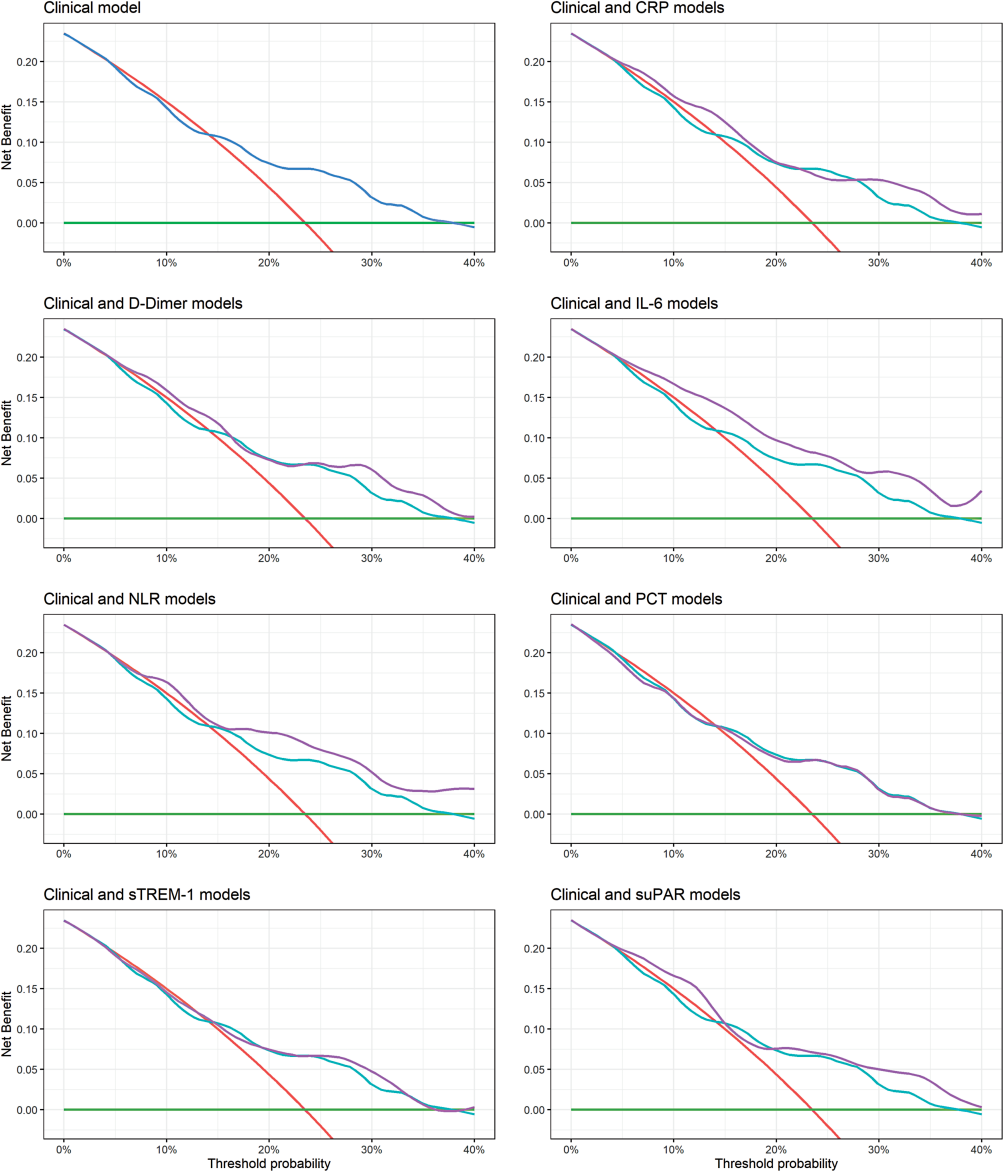
Decision curve analysis for each model in the validation cohort. The net benefit for each model is compared to an “admit-all” (*red line*) and “admit-none” (*green line*) approach, and each model containing a biochemical biomarker (*purple line*) is also compared to the model containing only clinical variables (*blue line*). A threshold probability of 5% indicates a scenario where the value of 1 TP (patient admitted who will subsequently require oxygen) is equivalent to 19 FPs (patients admitted who will not subsequently require oxygen). Abbreviations: CRP, C-reactive protein; FP, false positive; IL-6, interleukin 6; NLR, neutrophil-to-lymphocyte ratio; PCT, procalcitonin; sTREM-1, soluble triggering receptor expressed on myeloid cell-1; suPAR, soluble urokinase plasminogen activator receptor; TP, true positive.

We recognized that the relative value of a TP and FP, that is, admitted patients who would and would not subsequently require supplemental oxygen, was not fixed and would vary at different stages of the pandemic, reflecting bed pressures and/or capacity for follow-up [20]. Decision curve analyses accounting for this differential weighting suggest that the clinical model could provide utility (net benefit over an “admit-all” approach) at a threshold probability above 15% (ie, when the value of 1 TP is equal to ~7 FPs). Furthermore, the results indicate that models containing any 1 of IL-6, NLR, or suPAR could offer greater net benefit than the clinical model and extend the range of contexts in which a model might provide utility to include threshold probabilities above 5% (value of 1 TP is equal to 19 FPs; ie, when bed pressures are less critical). For the model containing IL-6, this higher net benefit appeared to be maintained across a range of plausible threshold probabilities (Figure 4).

## DISCUSSION

We report the development and temporal validation of 3 promising clinical prediction models to assist with the assessment of patients with moderate COVID-19. The models combine 3 simple parameters (age, sex, and SpO_2_) with measurement of a single biochemical biomarker (IL-6, NLR, or suPAR), quantifiable using commercially available rapid tests.

We included patients in whom there is clinical uncertainty as to whether admission is warranted, and adopted an analytical approach which acknowledged that the trade-offs inherent in this decision will vary at different stages of the pandemic and in different healthcare settings. We used specific systemic symptoms to define moderate severity disease rather than the WHO-CPS, recognizing, as did the scale’s original authors, that the lower end of the WHO-CPS is subjective [19]. Performance of any prediction model is sensitive to the prevalence of the outcome it aims to predict, and thus we hope our more objective study entry criteria will better standardize the outcome prevalence and facilitate model transportability; we followed the widely used ISARIC case report form to define symptoms to permit validation by other groups [33].

Our approach focused on quantifying the added value of host biomarkers. We recognize that laboratory tests carry an opportunity cost, especially when resources are limited. Although a model containing clinical parameters alone would be simpler to implement, our analyses indicate that inclusion of 1 biomarker test would allow use of the model in a broader range of contexts, including when bed pressures are less acute early in a COVID-19 surge.

Our models have face validity. All clinical and laboratory predictors have been implicated in the pathogenesis of COVID-19 [10, 17, 23, 25, 27, 29] Similar to others, we found that age and sex were not strongly associated with risk of deterioration, in contrast to their well-recognized association with COVID-19 mortality [23]. This underlines the importance of developing models for specific clinical use cases. Models developed to predict mortality are not necessarily appropriate to rule out less severe disease, just as models developed in well-resourced healthcare systems may not generalize to resource-limited settings [34].

The 3 biochemical biomarkers that demonstrate most promise in our study have biological plausibility. In addition to being a therapeutic target [35], raised IL-6 levels predict development of an oxygen requirement [27, 28] and, along with an elevated NLR, form part of the COVID-19-associated hyperinflammatory syndrome (cHIS) diagnostic criteria [36]. Elevated suPAR levels are associated with disease severity and progression in both moderate and severe COVID-19 [29, 37] and have been used for stratification into trials of immunomodulatory agents [38].

We addressed the limitations identified in other COVID-19 prognostic models by following the TRIPOD guidelines [18], and using a prospectively collected data set with minimal loss to follow-up and missing data [8]. Nevertheless, the small validation cohort (determined by the natural history of the pandemic in India) limits our ability to draw strong conclusions. Although the same models appeared superior in the different analyses we performed, further external validation is required before they can be recommended for use; we have published our full models (Supplementary Table 5; Supplementary Figure 5) to encourage independent validation.

No vaccinated individuals were included in the study. The models may require recalibration for use in vaccinated populations with lower baseline risk of progression to severe COVID-19. However, it is important to note that only 15/54 African countries met the WHO target of vaccinating 10% of their population by the end of September 2021 [39]. An estimated 55–70% vaccination coverage is required to achieve herd immunity for a vaccine with 90% efficacy [40]. Unfortunately, the timelines for adequate vaccination coverage in many LMICs are likely to be long.

In our context, corticosteroids were readily available and often self-prescribed or used off-license. Although steroid use was associated with some candidate predictors, it was not associated with the primary outcome and is therefore unlikely to have confounded the observed association (Supplementary Tables 7–8).

We selected oxygen requirement as our primary outcome as this reflects a clinically meaningful endpoint. We opted to use an SpO_2_/FiO_2 _< 400 for participants without documented hypoxia or tachypnoea prior to initiation of supplemental oxygen, as the threshold for oxygen therapy can be subjective and vary depending on available resources [19, 22]. It is unlikely that our outcome lacked sensitivity; only 1 participant who received supplemental oxygen did not meet the primary outcome. It may have lacked specificity (12 participants who met the primary outcome did not receive supplemental oxygen and calculation of FiO_2_ in nonventilated patients can overestimate pulmonary dysfunction) [41], but sensitivity would always be prioritized in a tool to inform community-based management. Furthermore, any outcome misclassification is likely to have reduced, rather than exaggerated, the prognostic performance of the candidate predictors and models [42].

Baseline Ct value was not associated with the risk of deterioration (Supplementary Table 9). In keeping with others, we found that seronegativity at enrollment was associated with an increased risk of deterioration (49/190 [25.8%] vs. 37/222 [16.7%]; χ^2^= 5.16; *P* = .023) [43, 44]. As rapid antibody tests are available this warrants further exploration, acknowledging that this is likely most relevant in patients without a history of previous COVID-19 illness or vaccination.

In conclusion, we present 3 clinical prediction models that could help clinicians to identify patients with moderate COVID-19 who are suitable for community-based management. The models address an unmet need in the COVID-19 care continuum. They are of particular relevance where resources are scarce and, if validated, would be practical for implementation. Routinely collected data from MSF medical facilities across 26 LMICs indicate that 54.4% (18 400/33 780) of patients presenting with clinically suspected COVID-19 between March 2020 and November 2021 who might be considered for admission, or 16.2% of all patients (18 400/113 455), would have been eligible for assessment using our models, illustrating the potential for widespread impact.

## Supplementary Data

Supplementary materials are available at *Clinical Infectious Diseases* online. Consisting of data provided by the authors to benefit the reader, the posted materials are not copyedited and are the sole responsibility of the authors, so questions or comments should be addressed to the corresponding author.

ciac224_suppl_Supplementary_Appendix

## Notes

***Acknowledgments*.** The authors are grateful to the members of the External Advisory Panel, led by Professor Nicholas J. White (MORU), for their advice and guidance throughout the study. They also acknowledge the support of Kundavaram P. P. Abhilash (CMC), O. C. Abraham (CMC), T. Balamugesh (CMC), Thambu David (CMC), Divya Dayanand (CMC), Biju George (CMC), Richa Gupta (CMC), Samuel G Hansdak (CMC), Ramya Iyadurai (CMC), Rajiv Karthik (CMC), Sharwar Kazmi (MSF), Mavuto Mukaka (MORU), Sowmya Sathyendra (CMC), Merylin Sebastian (CMC), Christopher Smith (LSHTM), Ramesh Vishwakarma (MORU), Sophie Yacoub (OUCRU), and Anand Zachariah (CMC).

***Author contributions*.** A. C., N. D., A. D., C. J., P. T., G. M. V., Y. L., and S. B. conceptualized the study. A. T. L. C. conducted the biomarker literature review. A. C., S. D., J. O., Y. L., and S. B. shortlisted the candidate biomarkers. P. G., K. G., D. B., R. K., S. K., A. L., A. M., Ve. K. S., J. S., and P. K. collected the clinical data. T. G., Sh. K., V. K., D. K., M. M., M. R. G., Sa. S., Vi. K. S., and S. B. were responsible for specimen processing and the laboratory assays. A. C., R. M., P. G., L. M., S. R. G., S. H., C. M., and N. W. curated the data. A. C., L. M., C. K., and S. B. wrote and approved the statistical analysis plan. A. C., R. M., L. M., and C. K. did the formal analysis. C. M., J. T., and N. W. were responsible for study monitoring. A. C. wrote the original draft of the manuscript. A. C., R. M., P. G., K. G., D. B., L. M., A. T. L. C., N. D., S. D., A. D., T. G., S. R. G., S. H., C. J., R. K., Sa. K., Sh. K., V. K., D. K., A. L., A. M., C. M., M. M., J. O., M. R. G., Sa. S., Ve. K. S., Vi. K. S., J. S., Su. S., J. T., P. T., A. M. G. V., N. W., P. K., G. M. V., C. K., Y. L., and S. B. reviewed, edited, and approved the manuscript. A. C., L. M., C. K., and S. B. verified the underlying data.

***Data sharing.*** De-identified, individual participant data from this study will be available to researchers whose proposed purpose of use is approved by the data access committees at Médecins Sans Frontières and the Mahidol-Oxford Tropical Medicine Research Unit. Inquiries or requests for the data may be sent to data.sharing@london.msf.org and datasharing@tropmedres.ac. Researchers interested in accessing biobanked samples should contact the corresponding authors who will coordinate with the respective institutions.

***Financial support.*** The PRIORITISE (Prognostication of Oxygen Requirement in Patients with Non-severe SARS-CoV-2 Infection) study was funded by MSF, India, who maintained a sponsor/investigator role for the study. The Wellcome Trust provides core funding to the Mahidol-Oxford Tropical Medicine Research Unit in Bangkok (grant numbers 220211, 215604/Z/19/Z), which supported the design, monitoring, and analysis of the study. C. K. is supported by a Wellcome Trust/Royal Society Sir Henry Dale Fellowship (grant number 211182/Z/18/Z). For the purpose of open access, the author has applied a CC BY public copyright license to any Author Accepted Manuscript version arising from this submission.

***Potential conflicts of interests.*** J. O. and S. D. report being employed by FIND, the global alliance for diagnostics (https://www.finddx.org/), an organization dedicated to advancing the use of diagnostic tools. All other authors report no potential conflicts. All authors have submitted the ICMJE Form for Disclosure of Potential Conflicts of Interest. Conflicts that the editors consider relevant to the content of the manuscript have been disclosed.
