# Supplementary material for: Facilitating Safe Discharge Through Predicting Disease Progression in Moderate Coronavirus Disease 2019 (COVID-19): A Prospective Cohort Study to Develop and Validate a Clinical Prediction Model in Resource-Limited Settings
Source: Clin Infect Dis. 2022 Mar 21;75(1):e368–79. doi: 10.1093/cid/ciac224 (PMC9129107; doi:10.1093/cid/ciac224)
Supplement: ciac224_suppl_Supplementary_Appendix [file ciac224_suppl_supplementary_appendix.docx]

**Facilitating safe discharge through predicting disease progression in moderate COVID-19: a prospective study to develop and externally validate a clinical prediction model in resource-limited settings**

**SUPPLEMENTARY APPENDIX**

**CONTENTS PAGE**

1. **Case report form** 2
2. **Results of literature review** 11
3. **Examples of rapid tests for candidate biomarkers** 13
4. **Proportion of missing data for candidate predictors** 14
5. **Baseline characteristics of development and validation cohorts** 15
6. **Levels of supplemental oxygen support for participants who met the primary outcome** 19
7. **Kaplan-Meier curves to indicate time to meeting the primary outcome** 20
8. **Univariate relationships between candidate predictors and the primary outcome** 21
9. **Unadjusted and adjusted associations between candidate predictors and primary outcome** 22
10. **Final model equations to illustrate how to use the clinical prediction rules** 24
11. **Classification measures for the different models in the validation cohort** 26
12. **Sensitivity and specificity for the different models in the validation cohort** 28
13. **Association of corticosteroid use with primary outcome** 29
14. **Association of corticosteroid use with candidate predictors** 30
15. **Association of RT-PCR Ct values with primary outcome** 31
16. **STROBE checklist** 32
17. **TRIPOD checklist** 34

**PRIORITISE**

**PR**ognost**I**cation of **O**xygen **R**equ**I**remen**T** **I**n non-severe **S**ARS-CoV-2 inf**E**ction

| **ELIGIBILITY CHECK** | | | | | | |
| --- | --- | --- | --- | --- | --- | --- |
| Inclusion criteria (all should be “Yes”) | | | | **YES** | | **NO** |
| Aged ≥ 18 years, and willing and able to give informed consent and comply with study procedures | | | | 🌕 | | 🌕 |
| RT-PCR or antigen test positive for SARS-CoV-2 during current illness^^[[1]](#footnote-1)^^ | | | | 🌕 | | 🌕 |
| Systemic manifestation of SARS-CoV-2 infection defined as:  Breathing difficulty  **OR**  History of fever during current illness **AND** chest pain OR abdominal pain OR loose stool OR severe myalgia | | | | 🌕 | | 🌕 |
| Exclusion criteria (all should be “No”) | | | | **YES** | | **NO** |
| Requires supplemental oxygen^[[2]](#footnote-2)^ or mechanical ventilation (invasive / non-invasive) at presentation | | | | 🌕 | | 🌕 |
| Laboratory confirmed SARS-CoV-2 infection (virological or serological) during a previous illness episode | | | | 🌕 | | 🌕 |
|  | | |  | |  | |
| Participant’s eligibility confirmed? | Yes 🌕 | No 🌕 | | | | |
| Participant study ID | \|___\|___\|___\|-\|___\|___\|___\|___\| | | | | | |
| Enrolled by (initials) | \|___\|___\| | | | | | |
|  | | |  | |  | |

**NOTES**

🌕 = Single selection permitted

□ = Multiple selections permitted

| **ENROLMENT** | | | | | | | **DATE:** \|___\|___\|-\|___\|___\|-\|___\|___\|___\|___\| | | | | | | | | | | | | | | | | | | | |
| --- | --- | --- | --- | --- | --- | --- | --- | --- | --- | --- | --- | --- | --- | --- | --- | --- | --- | --- | --- | --- | --- | --- | --- | --- | --- | --- |
| **PARTICIPANT BACKGROUND** | | | | | | | | | | | | | | | | | | | |  | | | | | | |
| **Age** | \|___\|___\| years | | | | | | | | **Sex** | | | Male 🌕 Female 🌕 | | | | | **If Female: Pregnant** | | | | Yes 🌕 | | | | | No 🌕 |
| **Location of residence** | | | | | | _________________________________________________________________________ | | | | | | | | | | | | | | | | | | | | |
|  | | |  | | | | | | | | | | | | | | | |  |  | | | | | | |
| **VITAL SIGNS** | | | | | **Time of measurement** \|___\|___\|:\|___\|___\| | | | | | | | | | | | | | | | | | | | | | |
| **Respiratory rate** | | \|___\|___\| bpm | | | | | | | | | **Oxygen saturation (room air)** | | | | \|___\|___\|___\| % | | | | | | | | | | | |
| **Heart rate** | | \|___\|___\|___\| bpm | | | | | | | | | **Blood Pressure** | | | | \|___\|___\|___\| / \|___\|___\|___\| mmHg | | | | | | | | | | | |
| **Axillary temperature** | | \|___\|___\|___\|**.**\|___\|°F | | | | | | | | | **Mental status** | | | | | Alert 🌕 | | | Voice 🌕 | Pain 🌕 | | | Unresponsive 🌕 | | | |
| **Weight** | | \|___\|___\|___\|**.**\|___\| kg | | | | | | | | | **Height** | | | | | \|___\|___\|___\| cm | | | | | | | | | | |
|  | | | |  | | | | | | |  | | | |  | | |  | |  | |  | | | | |
| **PRESENTING CLINICAL SYNDROME** | | | | | | | | | | | | | | | | | | | | | | | | | | |
|  | | | | | | | | **Yes** | | **No** | | |  | | | | | | | **Yes** | | | | **No** | | |
| **History of fever** | | | | | | | | 🌕 | | 🌕 | | | **Headache** | | | | | | | 🌕 | | | | 🌕 | | |
| **Cough** | | | | | | | | 🌕 | | 🌕 | | | **Altered consciousness** | | | | | | | 🌕 | | | | 🌕 | | |
| **Sore throat** | | | | | | | | 🌕 | | 🌕 | | | **Seizures** | | | | | | | 🌕 | | | | 🌕 | | |
| **Runny nose** | | | | | | | | 🌕 | | 🌕 | | | **Abdominal pain** | | | | | | | 🌕 | | | | 🌕 | | |
| **Ear pain** | | | | | | | | 🌕 | | 🌕 | | | **Nausea / vomiting** | | | | | | | 🌕 | | | | 🌕 | | |
| **Wheezing** | | | | | | | | 🌕 | | 🌕 | | | **Diarrhoea** | | | | | | | 🌕 | | | | 🌕 | | |
| **Chest pain** | | | | | | | | 🌕 | | 🌕 | | | **Conjunctivitis** | | | | | | | 🌕 | | | | 🌕 | | |
| **Muscle aches** | | | | | | | | 🌕 | | 🌕 | | | **Skin rash** | | | | | | | 🌕 | | | | 🌕 | | |
| **Joint pain (arthralgia)** | | | | | | | | 🌕 | | 🌕 | | | **Skin ulcers** | | | | | | | 🌕 | | | | 🌕 | | |
| **Fatigue / malaise** | | | | | | | | 🌕 | | 🌕 | | | **Lymphadenopathy** | | | | | | | 🌕 | | | | 🌕 | | |
| **Shortness of breath** | | | | | | | | 🌕 | | 🌕 | | | **Loss of smell** | | | | | | | 🌕 | | | | 🌕 | | |
| **Lower chest indrawing** | | | | | | | | 🌕 | | 🌕 | | | **Loss of taste** | | | | | | | 🌕 | | | | 🌕 | | |
| **Anorexia / loss of appetite** | | | | | | | | 🌕 | | 🌕 | | | **Other** | | | | | | | _____________________ | | | | | | |
| **Onset of first symptom** | | | | | | | | \|___\|___\| days | | | | | | **Impact on daily activities** | | | | | | Yes 🌕 | | | | | No 🌕 | |

| **CURRENT / RECENT MEDICATIONS** | | | | | | | | | | | | | | | | | | | | | | | |
| --- | --- | --- | --- | --- | --- | --- | --- | --- | --- | --- | --- | --- | --- | --- | --- | --- | --- | --- | --- | --- | --- | --- | --- |
| **New medications taken in last 14 days** | Steroids □ | | | | | Azithromycin □ | | | | | Remdesivir □ | | | | | | | Lopinavir / Ritonavir (Kaletra) □ | | | | | |
|  | Hydroxychloroquine / Chloroquine □ | | | | | | | | | | | | None □ | | | | Other □ __________________ | | | | | | |
|  | **If Other, please specify:** | | | | | | | | | Intravenous □ | | | | Intramuscular □ | | | | | | | Oral □ | | |
|  |  | | | | | | | | | | | | | | | | | | | | | | |
| **PAST MEDICAL HISTORY** | | | | | | | | | | | | | | | | | | | | | | | |
| **Current smoker** | Yes 🌕 | | | | No 🌕 | | | **If No, please specify**: | | | | | | | Former Smoker 🌕 | | | | | | | Never smoked 🌕 | |
| **Known comorbidities** | Yes 🌕 | | | | No 🌕 | | |  | | | | | | | | | | | | | | | |
| **If Yes:** | **Yes** | | | **No** | | | | |  | | | | | | | | | | **Yes** | | | | **No** |
| **Cardiovascular disease** | 🌕 | | | 🌕 | | | | | **Chronic kidney disease** | | | | | | | | | | 🌕 | | | | 🌕 |
| **Diabetes** | 🌕 | | | 🌕 | | | | | **Chronic neurological disorder** | | | | | | | | | | 🌕 | | | | 🌕 |
| **Hypertension** | 🌕 | | | 🌕 | | | | | **HIV** | | | | | | | | | | 🌕 | | | | 🌕 |
| **Malignant neoplasm** | 🌕 | | | 🌕 | | | | | **Other immunosuppression** | | | | | | | | | | 🌕 | | | | 🌕 |
| **Chronic lung disease** | 🌕 | | | 🌕 | | | | | **Liver disease** | | | | | | | | | | 🌕 | | | | 🌕 |
| **Asthma** | 🌕 | | | 🌕 | | | | | **Active TB** | | | | | | | | | | 🌕 | | | | 🌕 |
| **Other** | _________________________________________________________________________ | | | | | | | | | | | | | | | | | | | | | | |
|  | | | | | | | | | | | | | | | | | | | | | | | |
| **ENROLMENT SAMPLES** | |  | | | | | | | | | | | | | | | | | | | | | |
| **Venous blood sample** | Collected 🌕 | | | | | | Not collected 🌕 | | | | | **Time of collection** \|___\|___\|:\|___\|___\| | | | | | | | | | | | |
| **Respiratory swab** | Collected 🌕 | | | | | | Not collected 🌕 | | | | | | | | | | | | | | | | |
|  |  | | | | | |  | | | | | | | | | | | | | | | | |
| **HEALTH WORKER DECISION** | | | | | | | | | | | | | | | | | | | | | | | |
| **Admission decision** | Admit 🌕 | | Not admit 🌕 | | | | | **If admitted, reason:** | | | | | | | | Clinical 🌕 | | | | Public health / isolation 🌕 | | | |

| **ENROLMENT SAMPLES** | | | | | | |
| --- | --- | --- | --- | --- | --- | --- |
| **Date of full blood count collection** | | \|___\|___\|-\|___\|___\|-\|___\|___\|___\|___\| | | | | |
| **Leukocyte count** | \|___\|___\|.\|___\|x10^3^/µL | | **Lymphocyte count** | | \|___\|___\|.\|___\|x10^3^/µL | |
| **Neutrophil count** | \|___\|___\|.\|___\|x10^3^/µL | | **Platelet count** | | \|___\|___\|___\|x10^3^/µL | |
| **Date of respiratory swab collection** | | \|___\|___\|-\|___\|___\|-\|___\|___\|___\|___\| | | | | |
| **Respiratory swab** (SARS-CoV-2 RT-PCR) | | | Positive 🌕 | Negative 🌕 | |  |
|  |  |  | **If positive:** C_t_ value \|___\|___\|.\|___\| | | | |

| **DAILY FU** | **DAY 0** | | | | | **DAY 1** | | | | | **DAY 2** | | | | |
| --- | --- | --- | --- | --- | --- | --- | --- | --- | --- | --- | --- | --- | --- | --- | --- |
| **Date of follow-up** | \|__\|__\| /\|__\|__\| / 20 \|__\|__\| | | | | | \|__\|__\| /\|__\|__\| / 20 \|__\|__\| | | | | | \|__\|__\| /\|__\|__\| / 20 \|__\|__\| | | | | |
| **Discharged** | YES | | | NO | | YES | | | NO | | YES | | | NO | |
| **Alive** | YES | | | NO | | YES | | | NO | | YES | | | NO | |
| **Ventilated** | YES | | | NO | | YES | | | NO | | YES | | | NO | |
| **Sx resolved** | YES | | | NO | | YES | | | NO | | YES | | | NO | |
| **Supplemental O₂** | YES | | | NO | | YES | | | NO | | YES | | | NO | |
| ***First RR > 30** | \|__\|__\| bpm | | Time: \|__\|__\|:\|__\|__\| | | | \|__\|__\| bpm | | Time: \|__\|__\|:\|__\|__\| | | | \|__\|__\| bpm | | Time: \|__\|__\|:\|__\|__\| | | |
| ***Highest RR >30** | \|__\|__\| bpm | | Time: \|__\|__\|:\|__\|__\| | | | \|__\|__\| bpm | | Time: \|__\|__\|:\|__\|__\| | | | \|__\|__\| bpm | | Time: \|__\|__\|:\|__\|__\| | | |
| ***First SpO₂ ≤ 93%** | \|__\|__\| % | | Time: \|__\|__\|:\|__\|__\| | | | \|__\|__\| % | | Time: \|__\|__\|:\|__\|__\| | | | \|__\|__\| % | | Time: \|__\|__\|:\|__\|__\| | | |
| ***Lowest SpO₂ ≤ 93%** | \|__\|__\| % | | Time: \|__\|__\|:\|__\|__\| | | | \|__\|__\| % | | Time: \|__\|__\|:\|__\|__\| | | | \|__\|__\| % | | Time: \|__\|__\|:\|__\|__\| | | |
| **Current medication** | 0. None 1.Steroids 2. Azithromycin 3. Remdesivir 4. Lopinavir/Ritonavir 5. Hydroxychloroquine/Chloroquine 6. Convalescent Plasma 7. Other | | | | | | | | | | | | | | |
|  | 0 1  2  3  4  5  6  7  Others______________________________ | | | | | 0 1  2  3  4  5  6  7  Others______________________________ | | | | | 0 1  2  3  4  5  6  7  Others______________________________ | | | | |
| ****O₂ delivery** | 1.NC 2. FM 3. Venturi 4. HFNO/NIV 5. Ventilated 6. None | | | | | | | | | | | | | | |
|  | 1  2  3  4  5  6 | 1  2  3  4  5  6 | | | 1  2  3  4  5  6 | 1  2  3  4  5  6 | 1  2  3  4  5  6 | | | 1  2  3  4  5  6 | 1  2  3  4  5  6 | 1  2  3  4  5  6 | | | 1  2  3  4  5  6 |
| ****Flow rate (L/min)** | \|__\|__\|.\|__\| | \|__\|__\|.\|__\| | | | \|__\|__\|.\|__\| | \|__\|__\|.\|__\| | \|__\|__\|.\|__\| | | | \|__\|__\|.\|__\| | \|__\|__\|.\|__\| | \|__\|__\|.\|__\| | | | \|__\|__\|.\|__\| |
| ****FiO_2_ (%)** | \|__\|__\|__\| | \|__\|__\|__\| | | | \|__\|__\|__\| | \|__\|__\|__\| | \|__\|__\|__\| | | | \|__\|__\|__\| | \|__\|__\|__\| | \|__\|__\|__\| | | | \|__\|__\|__\| |
| ****Lowest SpO₂ (%)** | \|__\|__\|__\| | \|__\|__\|__\| | | | \|__\|__\|__\| | \|__\|__\|__\| | \|__\|__\|__\| | | | \|__\|__\|__\| | \|__\|__\|__\| | \|__\|__\|__\| | | | \|__\|__\|__\| |
| ****Time** | \|_\|_\|:\|_\|_\| | \|_\|_\|:\|_\|_\| | | | \|_\|_\|:\|_\|_\| | \|_\|_\|:\|_\|_\| | \|_\|_\|:\|_\|_\| | | | \|_\|_\|:\|_\|_\| | \|_\|_\|:\|_\|_\| | \|_\|_\|:\|_\|_\| | | | \|_\|_\|:\|_\|_\| |

*If on supplemental O**₂**, document values prior to initiation; ** Only complete this section if participant has not already met the endpoint (RR > 30 or SpO**₂** ≤ 93%)

| ^1^**D7 FOLLOW-UP** | | | | | **Date of enrolment** \|___\|___\|-\|___\|___\|-\|___\|___\|___\|___\| | | | | | | | | | | | | | |
| --- | --- | --- | --- | --- | --- | --- | --- | --- | --- | --- | --- | --- | --- | --- | --- | --- | --- | --- |
| **Date of follow-up** \|___\|___\|-\|___\|___\|-\|___\|___\|___\|___\| | | | | | | | | | | | | In person 🌕 Telephone 🌕 | | | | | | |
| **Able to complete D7 follow-up** | | Yes 🌕 | | No 🌕 | **If no, specify reason:** | | | | | Uncontactable 🌕 | | | | | Refusal 🌕 | | | |
|  |  | |  | |  | | | | | | | | | | | | | |
| **Alive** | | Yes 🌕 | | No 🌕 | **If no, date of death** \|___\|___\|-\|___\|___\|-\|___\|___\|___\|___\| | | | | | | | | | | | | | |
| **Symptoms resolved** | | Yes 🌕 | | No 🌕 | **If yes, how many days ago** \|___\| | | | | | | | | | | | | | |
|  |  |  |  |  | **If no, symptoms worsening** | | | | | | | | ^2^ Yes 🌕 | | | No 🌕 | | |
| **Admitted (not study site)** | | Yes 🌕 | | No 🌕 | **If yes, name of facility** ___________________________________________ | | | | | | | | | | | | | |
|  |  |  |  |  | **If yes, access to medical records** | | | | | | | | ^3^ Yes 🌕 | | | No 🌕 | | |
| **Received oxygen** | | Yes 🌕 | | No 🌕 | **If yes, supplemental oxygen route** | | | | Nasal cannula 🌕 | | | | | FM / Venturi 🌕 | | | | HFNO / NIV 🌕 |
|  |  |  |  |  |  |  |  |  | Ventilated 🌕 | | | | | Unknown 🌕 | | | | |
|  |  |  |  |  | **If yes, date initiated** | | | \|___\|___\|-\|___\|___\|-\|___\|___\|___\|___\| | | | | | | | | | | |
|  |  |  |  |  | **If yes, location received** | | | | | ________________________________________ | | | | | | | | |
| **Medications taken since enrolment (not at study site)** | | Steroids □ | | | Hydroxychloroquine / Chloroquine □ | | | | | | Azithromycin □ | | | | | | Remdesivir □ | |
|  |  | Lopinavir / Ritonavir □ | | | | | Convalescent plasma □ | | | | | | None □ | | | Other □ ___________ | | |
|  |  | **If other, please specify:** | | | | Intravenous □ | | | | Intramuscular □ | | | | | Oral □ | | | |

**NOTES**

1. This form should be completed for all participants not admitted at the study site on D7
2. If symptoms worsening invite participant to re-attend study site and complete study site recall form
3. If participant admitted at another health facility and has access to their medical records, arrange to review them and complete an off-site admission form

| ^1^**D14 FOLLOW-UP** | | | | | **Date of enrolment** \|___\|___\|-\|___\|___\|-\|___\|___\|___\|___\| | | | | | | | | | | | | | | |
| --- | --- | --- | --- | --- | --- | --- | --- | --- | --- | --- | --- | --- | --- | --- | --- | --- | --- | --- | --- |
| **Date of follow-up** \|___\|___\|-\|___\|___\|-\|___\|___\|___\|___\| | | | | | | | | | | | | | In person 🌕 Telephone 🌕 | | | | | | |
| **Able to complete D14 follow-up** | | Yes 🌕 | | No 🌕 | **If no, specify reason:** | | | | | | Uncontactable 🌕 | | | | | Refusal 🌕 | | | |
|  |  | |  | |  | | | | | | | | | | | | | | |
| **Alive** | | Yes 🌕 | | No 🌕 | **If no, date of death** \|___\|___\|-\|___\|___\|-\|___\|___\|___\|___\| | | | | | | | | | | | | | | |
| **Symptoms resolved** | | Yes 🌕 | | ^2^ No 🌕 | **If yes, how many days ago** \|___\| | | | | | | | | | | | | | | |
| **Admitted (not study site)** | | Yes 🌕 | | No 🌕 | **If yes, name of facility** ___________________________________________ | | | | | | | | | | | | | | |
|  |  |  |  |  | **If yes, access to medical records** | | | | | | | | | ^3^ Yes 🌕 | | | No 🌕 | | |
| **Received oxygen** | | Yes 🌕 | | No 🌕 | **If yes, supplemental oxygen route** | | | | | Nasal cannula 🌕 | | | | | FM / Venturi 🌕 | | | | HFNO / NIV 🌕 |
|  |  |  |  |  |  |  |  |  |  | Ventilated 🌕 | | | | | Unknown 🌕 | | | | |
|  |  |  |  |  | **If yes, date initiated** | | | | \|___\|___\|-\|___\|___\|-\|___\|___\|___\|___\| | | | | | | | | | | |
|  |  |  |  |  | **If yes, location received** | | | | | | ________________________________________ | | | | | | | | |
| **Medications taken since D7 (not at study site)** | | Steroids □ | | | Hydroxychloroquine / Chloroquine □ | | | | | | | Azithromycin □ | | | | | | Remdesivir □ | |
|  |  | Lopinavir / Ritonavir □ | | | | | Convalescent plasma □ | | | | | | | None □ | | | Other □ ___________ | | |
|  |  | **If other, please specify:** | | | | Intravenous □ | | | | | Intramuscular □ | | | | | Oral □ | | | |
|  | |  | | | |  | | | | |  | | | | |  | | | |
| **CRF REVIEWED BY SITE PI / SUPERVISOR** | | | | | | Yes 🌕 | | No 🌕 | | | | | | | |  | | | |

**NOTES**

1. This form should be completed for all participants not admitted at the study site on D14
2. If symptoms still present invite participant to re-attend study site and complete study site recall form
3. If participant admitted at another health facility and has access to their medical records, arrange to review them and complete an off-site admission form

| **STUDY SITE RECALL D7** | | **Date of enrolment** \|___\|___\|-\|___\|___\|-\|___\|___\|___\|___\| | | | |
| --- | --- | --- | --- | --- | --- |
| **Date of recall** \|___\|___\|-\|___\|___\|-\|___\|___\|___\|___\| | | | | | **Time** \|___\|___\|:\|___\|___\| |
| **Respiratory rate > 30 bpm** | Yes 🌕 | | No 🌕 | Unknown 🌕 | **If yes, RR** \|___\|___\| bpm |
| **Oxygen saturation ≤ 93%** | Yes 🌕 | | No 🌕 | Unknown 🌕 | **If yes, SpO_2_** \|___\|___\| % |

| **STUDY SITE RECALL D14** | | | **Date of enrolment** \|___\|___\|-\|___\|___\|-\|___\|___\|___\|___\| | | | |
| --- | --- | --- | --- | --- | --- | --- |
| **Date of recall** \|___\|___\|-\|___\|___\|-\|___\|___\|___\|___\| | | | | | | **Time** \|___\|___\|:\|___\|___\| |
| **Respiratory rate > 30 bpm** | Yes 🌕 | No 🌕 | | Unknown 🌕 | **If yes, RR** \|___\|___\| bpm | |
| **Oxygen saturation ≤ 93%** | Yes 🌕 | No 🌕 | | Unknown 🌕 | **If yes, SpO_2_** \|___\|___\| % | |

| **OFF-SITE ADMISSION** | | **Date of enrolment** \|___\|___\|-\|___\|___\|-\|___\|___\|___\|___\| | | | | | | | | | | |
| --- | --- | --- | --- | --- | --- | --- | --- | --- | --- | --- | --- | --- |
| **Date of admission** \|___\|___\|-\|___\|___\|-\|___\|___\|___\|___\| | | | | | | | | | | | | |
| **Respiratory rate > 30 bpm prior to supplemental oxygen** | Yes 🌕 | | No 🌕 | | Unknown 🌕 | | | | | | | |
|  | **If yes, date first > 30** \|___\|___\|-\|___\|___\|-\|___\|___\|___\|___\| | | | | | | | | | | | |
|  | **If yes, first RR > 30** \|___\|___\| bpm | | | | | | | | **Time** \|___\|___\|:\|___\|___\| | | | |
| **Oxygen saturation ≤ 93% prior to supplemental oxygen** | Yes 🌕 | | No 🌕 | | Unknown 🌕 | | | | | | | |
|  | **If yes, date first ≤ 93** \|___\|___\|-\|___\|___\|-\|___\|___\|___\|___\| | | | | | | | | | | | |
|  | **If yes, first SpO_2_ ≤ 93** \|___\|___\| % | | | | | | | | | **Time** \|___\|___\|:\|___\|___\| | | |
| **If No to both of above:**  **For each mode / FiO_2_ combination** | **Date** | | | \|___\|___\|-\|___\|___\|-\|___\|___\|___\|___\| | | | | | | | | |
|  | **Mode** | | | NC 🌕 | | FM 🌕 | Venturi 🌕 | | | | HFNO / NIV 🌕 | Ventilated 🌕 |
|  | **FiO_2_** | | | ^1^ **Flow rate:** \|___\|___\|.\|___\| L / min  ^2^ **Percentage:** \|___\|___\|___\| % | | | | | | | | |
|  | **Lowest SpO_2_** | | | \|___\|___\|___\| % | | | | **Time** \|___\|___\|:\|___\|___\| | | | | |
| **If No to both of above:**  **For each mode / FiO_2_ combination** | **Date** | | | \|___\|___\|-\|___\|___\|-\|___\|___\|___\|___\| | | | | | | | | |
|  | **Mode** | | | NC 🌕 | | FM 🌕 | Venturi 🌕 | | | | HFNO / NIV 🌕 | Ventilated 🌕 |
|  | **FiO_2_** | | | ^1^ **Flow rate:** \|___\|___\|.\|___\| L / min  ^2^ **Percentage:** \|___\|___\|___\| % | | | | | | | | |
|  | **Lowest SpO_2_** | | | \|___\|___\|___\| % | | | | **Time** \|___\|___\|:\|___\|___\| | | | | |

**NOTES**

1. Flow rate should be completed if participant has received O2 via nasal cannula or face mask
2. Percentage should be completed if participant has received O2 via Venturi mask, high-flow nasal oxygen, or non-invasive or mechanical ventilation

**Results of literature review conducted to inform biomarker longlisting for PRIORITISE study.**

*Search strategy*

Date performed: 1 June 2020

("severe acute respiratory syndrome coronavirus 2"[Supplementary Concept] OR coronavirus*[tiab] OR SARS-CoV-2[tiab] OR 2019-nCoV[tiab] OR COVID-19[Supplementary Concept] OR COVID*[tiab] OR "Wuhan seafood market pneumonia"[tiab])

**AND**

(Biomarkers[MeSH] OR Biomarker*[tiab] OR "Biologic marker*"[tiab] OR "Biological marker*"[tiab] OR "Laboratory marker*"[tiab] OR "Lab marker*"[tiab]

**OR**

Prognosis[MeSH] OR Prognos*[tiab] OR ((Predict*[tiab] OR Risk*[tiab]) AND "Severity"[tiab] OR Outcome*[tiab] OR Progression[tiab] OR Deteriorat*[tiab])))
**AND**

2019/11[PDAT] : 9999[PDAT]

*Screening of results*


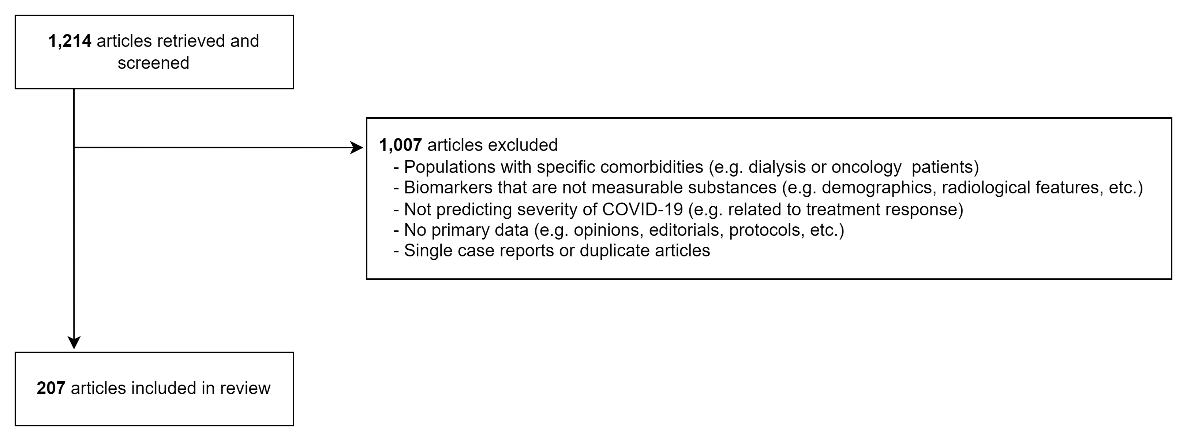
**Supplementary Figure 1. Screening of articles for inclusion.**

*Summary of main results*

**
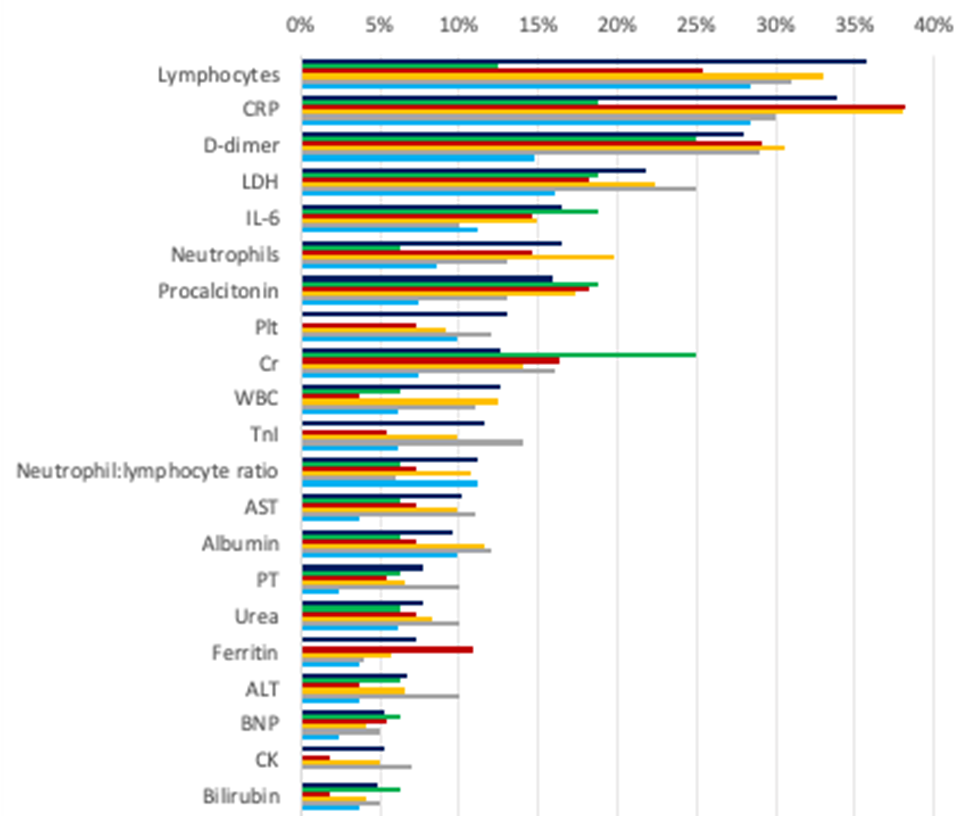
Supplementary Figure 2. Proportion of articles reporting statistically significant findings for different biochemical biomarkers in the prognostication of COVID-19.** Overall results (dark blue; n = 207) were broadly similar across different subgroups, including prospective studies (green; n = 16), studies outside China (red; n = 55); PCR/NGS-confirmed SARS-CoV-2 infection (yellow; n = 121); outcome determined after biomarker measurement i.e. prognostic vs. diagnostic (grey; n = 100), and studies with adjusted estimates (light blue; n = 81).

**Supplementary Table 1. Examples of commercially-available or late-stage development rapid quantitative tests for candidate biochemical biomarkers shortlisted for inclusion in the clinical prediction models.**

| **Biomarker** | **Manufacturer** | **Test characteristics** | | |
| --- | --- | --- | --- | --- |
|  |  | **Sample type** | **Sample volume** | **Turn-around-time** |
| CRP | NycoCard^TM^, Abbott | Whole blood, serum or plasma | 5uL | 3 minutes |
| D-dimer | RAMP, Response Biomedical | Whole blood | 75uL | 15 minutes |
| IL-6 | IL-6, Hotgen | Serum | 100uL | 15 minutes |
| NLR | WBC DIFF, HemoCue® | Whole blood | 10uL | 5 minutes |
| PCT | BRAHMS PCT direct, Roche | Whole blood | 20uL | 20 minutes |
| sTREM-1 | FIND, personal communication | Whole blood | TBC | TBC |
| suPAR | suPARnostic®, Virogates | Plasma | 10uL | 20 minutes |

**Supplementary Table 2. Number of participants with missing data for candidate predictor variables, stratified by cohort.**

| **Variable** | **Number missing** | | |
| --- | --- | --- | --- |
|  | **Development cohort**  (n = 257) | **Validation cohort**  (n = 166) | **Overall**  (n = 423) |
| Age (years) | 0/257 | 0/166 | **0/423** |
| Sex | 0/257 | 0/166 | **0/423** |
| SpO_2_ | 0/257 | 0/166 | **0/423** |
| CRP (mg/l) | 0/257 | 8/166 (4.8%) | **8/423 (1.9%)** |
| D-dimer (ng/ml) | 1/257 (0.4%) | 2/166 (1.2%) | **3/423 (0.7%)** |
| IL-6 (pg/ml) | 0/257 | 2/166 (1.2%) | **2/423 (0.5%)** |
| NLR | 10/257 (3.9%) | 1/166 (0.6%) | **11/423 (2.6%)** |
| PCT (ng/ml) | 0/257 | 2/166 (1.2%) | **2/423 (0.5%)** |
| sTREM-1 (pg/ml) | 0/257 | 2/166 (1.2%) | **2/423 (0.5%)** |
| suPAR (ng/ml) | 0/257 | 0/166 | **0/423** |

**Supplementary Table 3. Baseline characteristics of development and validation cohorts, stratified by primary outcome status, including additional details on presenting symptoms and reported comorbidities.** Details for presenting symptoms and reported comorbidities with prevalence ≥ 5% in any of the outcome groups for either the development or validation cohorts are reported. Data on other reported comorbidities (chronic neurological disorder, liver disease, active tuberculosis, malignant neoplasm, HIV, and other immunosuppression) and presenting symptoms (otalgia, wheeze, lower chest indrawing, altered consciousness, seizures, conjunctivitis, skin rash, skin ulcers, and lymphadenopathy) not reported as prevalence < 5% in all outcome groups. ^*^Missing data for continuous variables: BMI = 1; CRP = 8, D-dimer = 3, IL-6 = 2, NLR = 12; PCT = 2; sTREM-1 = 2; Ct value = 181. ^§^Different specimen collection procedures and PCR assays were used at each site (appendix p21). ^†^Seronegative defined as negative for both SARS-CoV-2 IgG and IgM antibodies. Median values (IQR) and p-values for Wilcoxon rank sum tests are reported for continuous variables. Fisher’s exact or Pearson’s Chi-squared test p-values are reported for categorical variables.

| **Baseline characteristic** | **DEVELOPMENT COHORT** | | | | **VALIDATION COHORT** | | | | **p-value** |
| --- | --- | --- | --- | --- | --- | --- | --- | --- | --- |
|  | **Overall**  (n = 257) | **Developed oxygen requirement** | | **p-value** | **Overall**  (n = 166) | **Developed oxygen requirement** | | **p-value** |  |
|  |  | **No**  (n = 207) | **Yes**  (n = 50) |  |  | **No**  (n = 127) | **Yes**  (n = 39) |  |  |
| **Demographics** | | | | | | | | | |
| Age (years) | 52.0  (40.0 to 61.0) | 52.0  (40.0 to 60.0) | 54.0  (42.2 to 62.0) | 0.3 | 54.0  (41.2 to 63.0) | 55.0  (41.5 to 63.0) | 54.0  (41.0 to 66.0) | 0.8 | 0.2 |
| Male sex | 185 / 257  (72%) | 144 / 207  (70%) | 41 / 50  (82%) | 0.079 | 101 / 166  (61%) | 76 / 127  (60%) | 25 / 39  (64%) | 0.6 | 0.017 |
| BMI (kg/m²) ^*^ | 26.0  (23.6 to 28.7) | 26.2  (23.8 to 28.8) | 25.8  (22.4 to 28.3) | 0.4 | 24.9  (23.4 to 27.6) | 24.8  (23.4 to 27.6) | 26.1  (23.7 to 27.6) | 0.7 | 0.071 |
| **Vital signs** | | | | | | | | | |
| Heart rate (bpm) | 88.0  (80.0 to 97.0) | 86.0  (79.0 to 96.0) | 90.0  (86.0 to 99.5) | 0.010 | 84.0  (74.0 to 92.0) | 84.0  (74.0 to 90.0) | 84.0  (77.0 to 94.0) | 0.4 | < 0.001 |
| Respiratory rate (bpm) | 22.0  (22.0 to 24.0) | 22.0  (22.0 to 24.0) | 22.0  (22.0 to 24.0) | 0.3 | 24.0  (22.0 to 24.0) | 22.0  (22.0 to 24.0) | 24.0  (22.0 to 24.0) | 0.12 | 0.006 |
| Oxygen saturation (%) | 98.0  (96.0 to 99.0) | 98.0  (97.0 to 99.0) | 96.0  (95.2 to 98.0) | < 0.001 | 98.0  (96.0 to 99.0) | 98.0  (96.0 to 99.0) | 96.0  (95.5 to 98.0) | 0.002 | 0.5 |
| Axillary temperature (°C) | 36.8  (36.4 to 37.1) | 36.7  (36.4 to 37.0) | 36.9  (36.5 to 37.2) | 0.045 | 36.9  (36.7 to 37.2) | 36.9  (36.7 to 37.2) | 37.0  (36.9 to 37.2) | 0.053 | < 0.001 |
| Systolic BP (mmHg) | 128.0  (116.0 to 138.0) | 128.0  (116.0 to 140.0) | 126.0  (118.0 to 134.8) | 0.7 | 121.0  (110.0 to 130.0) | 120.0  (110.0 to 130.0) | 122.0  (110.0 to 131.0) | > 0.9 | < 0.001 |
| Diastolic BP (mmHg) | 80.0  (72.0 to 88.0) | 80.0  (72.0 to 88.0) | 79.0  (70.0 to 88.0) | 0.3 | 76.0  (70.0 to 82.0) | 76.0  (70.0 to 82.0) | 74.0  (67.0 to 80.0) | 0.3 | < 0.001 |
| qSOFA score ≥ 2 | 13 / 257  (5.1%) | 9 / 207  (4.3%) | 4 / 50  (8.0%) | 0.3 | 16 / 166  (9.6%) | 10 / 127  (7.9%) | 6 / 39  (15%) | 0.2 | 0.069 |
| **Comorbidities** | | | | | | | | | |
| Current smokers | 10 / 257  (3.9%) | 8 / 207  (3.9%) | 2 / 50  (4.0%) | > 0.9 | 4 / 166  (2.4%) | 3 / 127  (2.4%) | 1 / 39  (2.6%) | > 0.9 | 0.4 |
| Reported comorbidity ^#^ | 165 / 257  (64%) | 128 / 207  (62%) | 37 / 50  (74%) | 0.11 | 117 / 166  (70%) | 91 / 127  (72%) | 26 / 39  (67%) | 0.6 | 0.2 |
| Cardiovascular disease | 21 / 252  (8.3%) | 16 / 203  (7.9%) | 5 / 49  (10%) | 0.6 | 19 / 166  (11%) | 15 / 127  (12%) | 4 / 39  (10%) | > 0.9 | 0.3 |
| Diabetes | 92 / 255  (36%) | 73 / 205  (36%) | 19 / 50  (38%) | 0.8 | 64 / 166  (39%) | 55 / 127  (43%) | 9 / 39  (23%) | 0.023 | 0.6 |
| Hypertension | 89 / 256  (35%) | 73 / 206  (35%) | 16 / 50  (32%) | 0.6 | 64 / 166  (39%) | 47 / 127  (37%) | 17 / 39  (44%) | 0.5 | 0.4 |
| Chronic lung disease | 3 / 252  (1.2%) | 3 / 203  (1.5%) | 0 / 49  (0%) | > 0.9 | 3 / 166  (1.8%) | 1 / 127  (0.8%) | 2 / 39  (5.1%) | 0.14 | 0.7 |
| Asthma | 16 / 252  (6.3%) | 13 / 203  (6.4%) | 3 / 49  (6.1%) | > 0.9 | 11 / 166  (6.6%) | 9 / 127  (7.1%) | 2 / 39  (5.1%) | > 0.9 | > 0.9 |
| Chronic kidney disease | 10 / 252  (4.0%) | 8 / 203  (3.9%) | 2 / 49  (4.1%) | > 0.9 | 9 / 166  (5.4%) | 5 / 127  (3.9%) | 4 / 39  (10%) | 0.2 | 0.5 |
| **Presenting illness** | | | | | | | | | |
| Symptom duration (days) | 6.0  (4.0 to 8.0) | 6.0  (4.0 to 8.0) | 5.5  (5.0 to 7.0) | 0.8 | 6.0  (4.0 to 8.0) | 6.0  (3.5 to 8.0) | 5.0  (4.0 to 7.0) | 0.5 | 0.10 |
| History of fever | 243 / 257  (95%) | 196 / 207  (95%) | 47 / 50  (94%) | 0.7 | 155 / 166  (93%) | 118 / 127  (93%) | 37 / 39  (95%) | > 0.9 | 0.6 |
| Breathlessness | 154 / 257  (60%) | 119 / 207  (57%) | 35 / 50  (70%) | 0.11 | 90 / 166  (54%) | 65 / 127  (51%) | 25 / 39  (64%) | 0.2 | 0.2 |
| Chest pain | 59 / 257  (23%) | 48 / 207  (23%) | 11 / 50  (22%) | 0.9 | 15 / 166  (9.0%) | 9 / 127  (7.1%) | 6 / 39  (15%) | 0.12 | < 0.001 |
| Abdominal pain | 35 / 257  (14%) | 32 / 207  (15%) | 3 / 50  (6.0%) | 0.080 | 15 / 166  (9.0%) | 12 / 127  (9.4%) | 3 / 39  (7.7%) | > 0.9 | 0.2 |
| Diarrhoea | 80 / 257  (31%) | 65 / 207  (31%) | 15 / 50  (30%) | 0.8 | 47 / 166  (28%) | 33 / 127  (26%) | 14 / 39  (36%) | 0.2 | 0.5 |
| Severe myalgia | 140 / 257  (54%) | 110 / 207  (53%) | 30 / 50  (60%) | 0.4 | 75 / 166  (45%) | 65 / 127  (51%) | 10 / 39  (26%) | 0.005 | 0.062 |
| Sore throat | 87 / 257  (34%) | 69 / 207  (33%) | 18 / 50  (36%) | 0.7 | 31 / 166  (19%) | 23 / 127  (18%) | 8 / 39  (21%) | 0.7 | < 0.001 |
| Rhinorrhoea | 49 / 257  (19%) | 42 / 207  (20%) | 7 / 50  (14%) | 0.3 | 19 / 166  (11%) | 14 / 127  (11%) | 5 / 39  (13%) | 0.8 | 0.037 |
| Cough | 205 / 257  (80%) | 160 / 207  (77%) | 45 / 50  (90%) | 0.045 | 144 / 166  (87%) | 109 / 127  (86%) | 35 / 39  (90%) | 0.5 | 0.065 |
| Arthralgia | 49 / 257  (19%) | 37 / 207  (18%) | 12 / 50  (24%) | > 0.9 | 2 / 166  (1.2%) | 2 / 127  (1.6%) | 0 / 39  (0%) | > 0.9 | < 0.001 |
| Fatigue | 190 / 257  (74%) | 153 / 207  (74%) | 37 / 50  (74%) | > 0.9 | 97 / 166  (58%) | 76 / 127  (60%) | 21 / 39  (54%) | 0.5 | < 0.001 |
| Anorexia | 69 / 257  (27%) | 52 / 207  (25%) | 17 / 50  (34%) | 0.2 | 14 / 166  (8.4%) | 12 / 127  (9.4%) | 2 / 39  (5.1%) | 0.5 | < 0.001 |
| Nausea or vomiting | 55 / 257  (21%) | 42 / 207  (20%) | 13 / 50  (26%) | 0.4 | 23 / 166  (14%) | 17 / 127  (13%) | 6 / 39  (15%) | 0.8 | 0.051 |
| Headache | 75 / 257  (29%) | 65 / 207  (31%) | 10 / 50  (20%) | 0.11 | 41 / 166  (25%) | 32 / 127  (25%) | 9 / 39  (23%) | 0.8 | 0.3 |
| Anosmia | 60 / 257  (23%) | 52 / 207  (25%) | 8 / 50  (16%) | 0.2 | 13 / 166  (7.8%) | 9 / 127  (7.1%) | 4 / 39  (10%) | 0.5 | < 0.001 |
| Aguesia | 60 / 257  (23%) | 51 / 207  (25%) | 9 / 50  (18%) | 0.3 | 19 / 166  (11%) | 14 / 127  (11%) | 5 / 39  (13%) | 0.8 | 0.002 |
| **Host biomarkers** | | | | | | | | | |
| CRP (mg/l) ^*^ | 24.4  (3.9 to 88.9) | 17.9  (2.8 to 85.4) | 62.5  (19.7 to 134.4) | < 0.001 | 58.1  (17.2 to 147.1) | 42.5  (12.3 to 111.9) | 95.8  (52.8 to 176.9) | < 0.001 | < 0.001 |
| D-dimer (ng/ml) ^*^ | 725.0  (382.4 to 1,466.4) | 640.6  (329.7 to 1,234.9) | 1,201.7  (679.9 to 2,307.0) | < 0.001 | 968.2  (620.7 to 1,599.0) | 918.8  (579.0 to 1,454.9) | 1,148.1  (829.5 to 3,200.2) | 0.009 | < 0.001 |
| IL-6 (pg/ml) ^*^ | 11.0  (4.9 to 36.2) | 8.7  (4.2 to 27.9) | 36.4  (18.4 to 70.7) | < 0.001 | 31.6  (13.9 to 63.0) | 24.4  (11.4 to 47.2) | 71.1  (39.4 to 98.9) | < 0.001 | < 0.001 |
| NLR ^*^ | 3.2  (1.9 to 4.9) | 2.9  (1.7 to 4.5) | 4.4  (3.2 to 7.2) | < 0.001 | 2.8  (1.8 to 5.4) | 2.5  (1.6 to 4.2) | 5.3  (2.7 to 7.0) | < 0.001 | 0.6 |
| PCT (ng/ml) ^*^ | 0.1  (0.1 to 0.2) | 0.1  (0.1 to 0.1) | 0.1  (0.1 to 0.2) | < 0.001 | 0.1  (0.1 to 0.2) | 0.1  (0.1 to 0.2) | 0.1  (0.1 to 0.3) | 0.3 | 0.004 |
| sTREM-1 (pg/ml) ^*^ | 378.0  (265.0 to 537.0) | 362.0  (259.0 to 522.0) | 424.5  (306.8 to 649.5) | 0.055 | 419.0  (285.0 to 596.8) | 389.0  (282.0 to 562.0) | 437.0  (349.0 to 660.8) | 0.13 | 0.2 |
| suPAR (ng/ml) | 4.2  (3.1 to 5.8) | 4.0  (2.9 to 5.5) | 5.4  (4.0 to 6.8) | < 0.001 | 4.1  (3.1 to 5.6) | 3.8  (2.9 to 5.1) | 5.5  (3.9 to 6.7) | < 0.001 | 0.9 |
| **Viral markers** | | | | | | | | | |
| Ct value ^*§^ | 26.0  (20.7 to 30.8) | 26.0  (20.6 to 30.1) | 26.4  (22.0 to 31.4) | 0.6 | 32.1  (28.3 to 36.2) | 32.8  (28.1 to 36.2) | 31.5  (28.4 to 36.0) | > 0.9 | < 0.001 |
| Seronegative ^†^ | 117 / 252  (46%) | 90 / 203  (44%) | 27 / 49  (55%) | 0.2 | 73 / 160  (46%) | 51 / 123  (41%) | 22 / 37  (59%) | 0.054 | 0.9 |

**Supplementary Table 4. Maximum level of supplemental oxygen support received by participants who met the primary outcome, stratified by cohort.** One participant (not included in this table) received supplemental oxygen via nasal cannula but did not meet the primary outcome as their SpO_2_/FiO_2_ remained above 400. ^*^One participant met the endpoint on the basis of being prescribed oxygen at another licensed medical facility; all other participants who met the endpoint did so on the basis of an SpO_2_ < 94% and/or SpO_2_/FiO_2_ < 400 and/or death.

|  | **Development**  (n = 257) | **Validation**  (n = 166) | **Overall**  (n=423) |
| --- | --- | --- | --- |
| **Number of participants meeting primary outcome** | 50 | 39 | 89 |
| **Deaths** | 2 | 9 | 11 |
| **Mechanical ventilation** | 1 | 1 | 2 |
| **Non-invasive ventilation** | 5 | 10 | 15 |
| **Supplemental oxygen via face mask and/or nasal cannula**^*^ | 32 | 17 | 49 |
| **No supplemental oxygen received** | 10 | 2 | 12 |

**Supplementary Figure 3. Kaplan-Meier survival curves indicating the time to meeting the primary outcome, stratified by cohort.** Coloured lines indicate survival probability for each cohort and shaded areas indicate 95% confidence intervals.

**
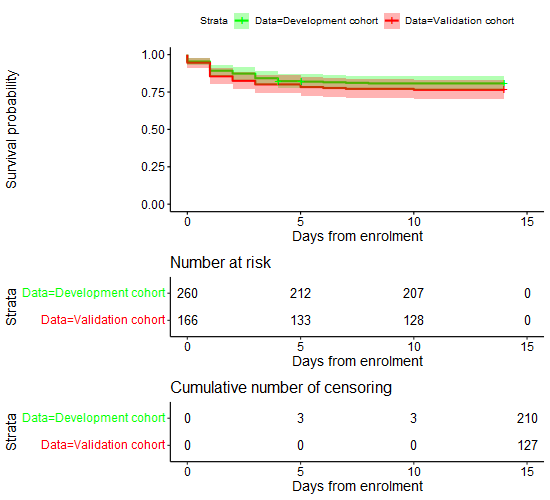
**

**
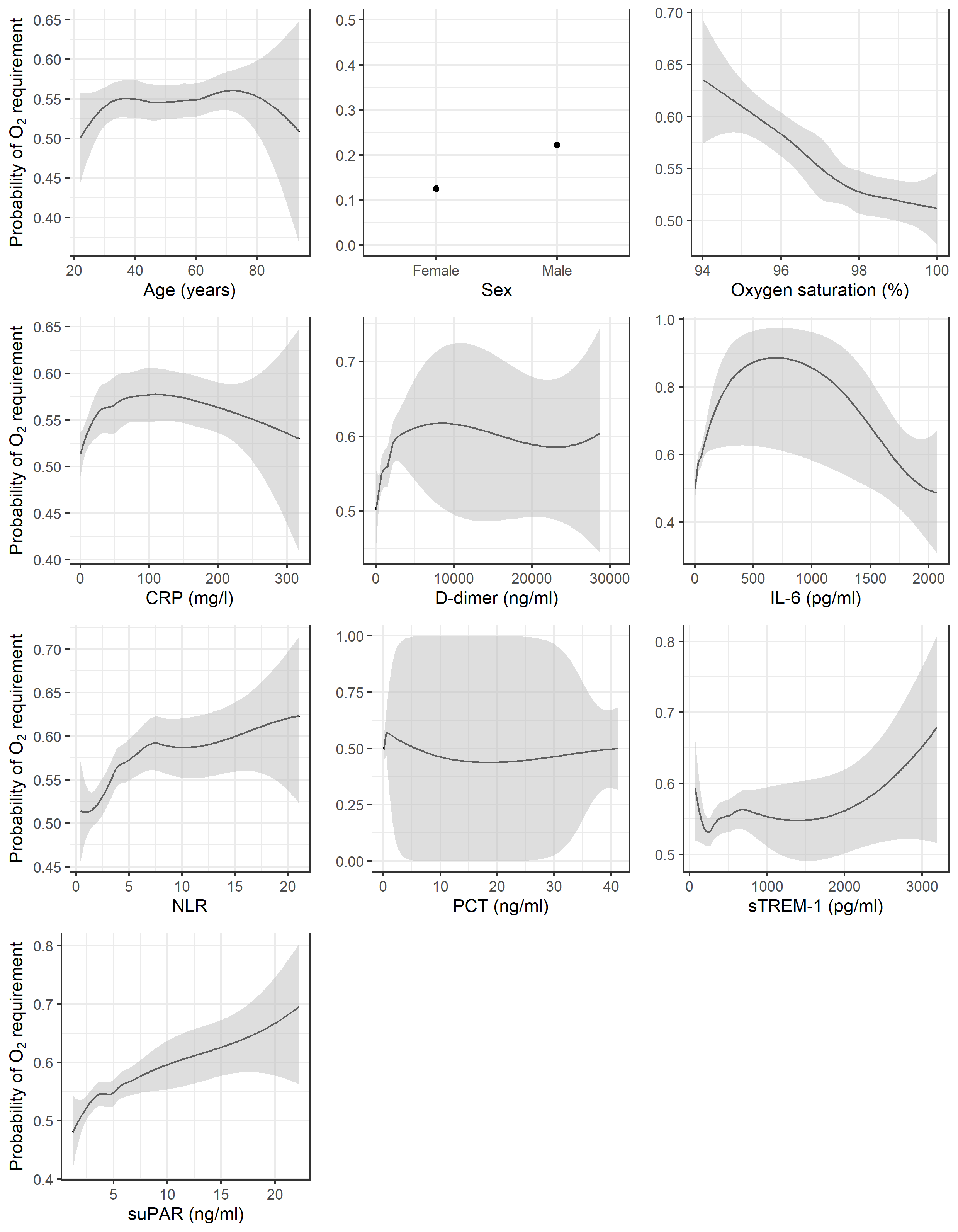
Supplementary Figure 4. Exploration of the relationship between candidate predictors and the outcome using a Lowess smoothing approach.**

**Supplementary Table 5. Unadjusted and adjusted associations between candidate predictors and primary outcome in each model in the development cohort**. 1,000 bootstrap samples were used to estimate 95% confidence intervals for the ridge regression coefficients. ^*^ Transformations used due to non-linear relationship between predictor and outcome: Log_10_ used for CRP and D-dimer; $\frac{1}{\sqrt{IL-6/100}}$ used for IL-6. As a result, odds ratios are not comparable between continuous predictors, as scales cannot be standardised. Odds ratios are expressed for a one unit increase in non-transformed continuous predictors (age, NLR, PCT, sTREM-1, suPAR) and a ten-fold increase in CRP or D-dimer.

| **Variable** | **Unadjusted**  **odds ratio**  **(95% CI)** | **Unadjusted**  **c-statistic**  **(95% CI)** | **Ridge regression coefficient**  **(95% CI)** | **Adjusted**  **odds ratio**  **(95% CI)** |
| --- | --- | --- | --- | --- |
| **Clinical model** | | | | |
| Intercept | NA | NA | 41.89  (23.14 to 67.65) | NA |
| Male sex | 1.99  (0.98 to 5.24) | NA | 0.31  (-0.30 to 1.22) | 1.37  (0.74 to 3.37) |
| Age (years) | 1.01  (0.99 to 1.03) | 0.55  (0.48 to 0.64) | 0.00  (-0.02 to 0.02) | 1.00  (0.98 to 1.02) |
| SpO_2_ | **0.56**  **(0.43 to 0.71)** | 0.72  (0.64 to 0.80) | -0.45  (-0.71 to -0.26) | **0.64**  **(0.49 to 0.77)** |
| **CRP model** | | | | |
| Intercept | NA | NA | 40.47  (21.51 to 66.25) | NA |
| Male sex | NA | NA | 0.16  (-0.56 to 1.03) | 1.18  (0.57 to 2.79) |
| Age (years) | NA | NA | -0.00  (-0.03 to 0.02) | 1.00  (0.97 to 1.02) |
| SpO_2_ | NA | NA | -0.44  (-0.71 to -0.24) | **0.64**  **(0.49 to 0.78)** |
| CRP (mg/l) ^*^ | **2.24**  **(1.59 to 3.52)** | 0.66  (0.58 to 0.73) | 0.54  (0.23 to 0.98) | **1.71**  **(1.26 to 2.65)** |
| **D-dimer model** | | | | |
| Intercept | NA | NA | 38.15  (22.19 to 67.18) | NA |
| Male sex | NA | NA | 0.34  (-0.29 to 1.26) | 1.40  (0.75 to 3.54) |
| Age (years) | NA | NA | -0.00  (-0.03 to 0.02) | 1.00  (0.97 to 1.02) |
| SpO_2_ | NA | NA | -0.44  (-0.75 to -0.28) | **0.65**  **(0.47 to 0.76)** |
| D-dimer (ng/ml) ^*^ | **3.32**  **(1.85 to 6.89)** | 0.67  (0.60 to 0.75) | 0.98  (0.47 to 1.88) | **2.67**  **(1.60 to 6.54)** |
| **IL-6 model** | | | | |
| Intercept | NA | NA | 39.92  (24.84 to 64.91) | NA |
| Male sex | NA | NA | 0.11  (-0.64 to 1.02) | 1.11  (0.53 to 2.76) |
| Age (years) | NA | NA | -0.01  (-0.03 to 0.02) | 0.99  (0.97 to 1.02) |
| SpO_2_ | NA | NA | -0.41  (-0.68 to -0.25) | **0.66**  **(0.51 to 0.78)** |
| IL-6 (pg/ml) ^*^ | **0.53**  **(0.39 to 0.65)** | 0.75  (0.68 to 0.82) | -0.44  (-0.66 to -0.25) | **0.65**  **(0.52 to 0.78)** |
| **NLR model** | | | | |
| Intercept | NA | NA | 38.27  (19.93 to 66.20) | NA |
| Male sex | NA | NA | 0.21  (-0.45 to 0.98) | 1.24  (0.63 to 2.65) |
| Age (years) | NA | NA | -0.00  (-0.03 to 0.02) | 1.00  (0.97 to 1.02) |
| SpO_2_ | NA | NA | -0.41  (-0.71 to -0.22) | **0.66**  **(0.49 to 0.80)** |
| NLR | **1.15**  **(1.07 to 1.27)** | 0.69  (0.61 to 0.77) | 0.10  (0.04 to 0.20) | **1.10**  **(1.04 to 1.22)** |
| **PCT model** | | | | |
| Intercept | NA | NA | 41.03  (1.03 to 66.59) | NA |
| Male sex | NA | NA | 0.32  (-0.29 to 1.19) | 1.37  (0.75 to 3.28) |
| Age (years) | NA | NA | 0.00  (-0.02 to 0.02) | 1.00  (0.98 to 1.02) |
| SpO_2_ | NA | NA | -0.44  (-0.71 to -0.03) | **0.64**  **(0.49 to 0.97)** |
| PCT (ng/ml) | 0.96  (0.80 to 1.74) | 0.66  (0.58 to 0.74) | -0.03  (-0.03 to 1.28) | 0.97  (0.97 to 3.60) |
| **sTREM-1 model** | | | | |
| Intercept | NA | NA | 42.55  (22.26 to 70.09) | NA |
| Male sex | NA | NA | 0.28  (-0.35 to 1.11) | 1.33  (0.71 to 3.02) |
| Age (years) | NA | NA | 0.00  (-0.02 to 0.02) | 1.00  (0.98 to 1.02) |
| SpO_2_ | NA | NA | -0.46  (-0.746 to -0.25) | **0.63**  **(0.48 to 0.78)** |
| sTREM-1 (pg/ml) | 1.00  (1.00 to 1.00) | 0.58  (0.48 to 0.68) | 0.00  (-0.00 to 0.00) | 1.00  (1.00 to 1.00) |
| **suPAR model** | | | | |
| Intercept | NA | NA | 38.82  (21.24 to 66.61) | NA |
| Male sex | NA | NA | 0.38  (-0.24 to 1.32) | 1.46  (0.79 to 3.74) |
| Age (years) | NA | NA | -0.00  (-0.03 to 0.02) | 1.00  (0.98 to 1.02) |
| SpO_2_ | NA | NA | -0.42  (-0.71 to -0.24) | **0.65**  **(0.49 to 0.79)** |
| suPAR (ng/ml) | **1.20**  **(1.11 to 1.36)** | 0.68  (0.61 to 0.76) | 0.14  (0.06 to 0.25) | **1.15**  **(1.06 to 1.29)** |

**Supplementary Figure 5. Final model equations to illustrate how the clinical prediction rules can be used to calculate the predicted probability of progression to a supplemental oxygen requirement for patients presenting with moderate COVID-19.** To maximise use of the available information all continuous predictors have been maintained as continuous variables and hence the clinical prediction rules would be most easily used with the help of a simple computer, for example as part of a web-browser-based or mobile application.

| $\Pr\left( \boldsymbol{Oxygen requirement} \right)\boldsymbol{=}\frac{\boldsymbol{e}^{\boldsymbol{LP}}}{\boldsymbol{1+}\boldsymbol{e}^{\boldsymbol{LP}}}$  where LP is the linear predictor  The LP predictor should be estimated for each model using the following equations:  **IL-6 model**  $LP\left( IL6 model \right)=39.92+\left\{ \begin{aligned} 0 if female \\ 0.11 if male \end{aligned}- \right. 0.007*age-0.41*SpO_{2}-0.44*\frac{1}{\sqrt{(IL6)/100}}$  **NLR model**  $LP\left( NLR model \right)=38.27+\left\{ \begin{aligned} 0 if female \\ 0.21 if male \end{aligned}- \right. 0.002*age-0.41*SpO_{2}+0.10*NLR$  **suPAR model**  $LP\left( suPAR model \right)=38.82+\left\{ \begin{aligned} 0 if female \\ 0.38 if male \end{aligned}- \right. 0.002*age-0.42*SpO_{2}+0.14*suPAR$  **Clinical model**  $LP\left( Clinical model \right)=41.89+\left\{ \begin{aligned} 0 if female \\ 0.31 if male \end{aligned}+ \right. 0.003*age-0.45*SpO_{2}$  **CRP model**  $LP\left( CRP model \right)=40.47+\left\{ \begin{aligned} 0 if female \\ 0.16 if male \end{aligned}- \right. 0.002*age-0.44*SpO_{2}+0.54*{log}_{10}\left( CRP \right)$  **D-dimer model**  $LP\left( D dimer \right)=38.15+\left\{ \begin{aligned} 0 if female \\ 0.34 if male \end{aligned}- \right. 0.002*age-0.44*SpO_{2}+0.98*{log}_{10}\left( D dimer \right)$  **PCT model**  $LP\left( PCT model \right)=41.03+\left\{ \begin{aligned} 0 if female \\ 0.32 if male \end{aligned}+ \right. 0.003*age-0.44*SpO_{2}-0.03*PCT$  **sTREM-1 model**  $LP\left( sTREM1 model \right)=42.55+\left\{ \begin{aligned} 0 if female \\ 0.28 if male \end{aligned}+ \right. 0.0003*age-0.46*SpO_{2}+0.0006*sTREM 1$  Note:  $\exp$ is the exponential function |
| --- |

**Supplementary Table 6. Sensitivity, specificity, negative predictive value and positive predictive value for each model at different cut-offs in the validation cohort**. A cut-off of 0.1 reflects a management strategy in which any patient with a predicted risk of requiring oxygen ≥ 10% is admitted.

| **CUT-OFF** | **Sensitivity**  **(95% CI)** | **Specificity**  **(95% CI)** | **Negative likelihood ratio**  **(95% CI)** | **Positive**  **likelihood ratio**  **(95% CI)** | **Negative predictive value**  **(95% CI)** | **Positive**  **predictive value**  **(95% CI)** |
| --- | --- | --- | --- | --- | --- | --- |
| **Clinical model** | | | | | | |
| 0.1 | 89.7  (75.8 to 97.1) | 25.2  (17.9 to 33.7) | 0.41  (0.15 to 1.08) | 1.20  (1.04 to 1.39) | 88.9  (73.9 to 96.9) | 26.9  (19.5 to 35.4) |
| 0.15 | 76.9  (60.7 to 88.9) | 40.9  (32.3 to 50.2) | 0.56  (0.31 to 1.04) | 1.30  (1.04 to 1.63) | 85.3  (73.8 to 93.0) | 28.6  (20.2 to 38.2) |
| 0.20 | 61.5  (44.6 to 76.6) | 62.9  (53.9 to 71.4) | 0.61  (0.40 to 0.93) | 1.66  (1.19 to 2.33) | 84.2  (75.3 to 90.9) | 33.8  (23.0 to 46.0) |
| **IL-6 model** | | | | | | |
| 0.1 | 100  (90.9 to 100) | 21.3  (14.5 to 29.4) | 0  (NA) | 1.27  (1.16 to 1.39) | 100  (87.2 to 100) | 28.1  (20.8 to 36.3) |
| 0.15 | 92.3  (79.1 to 98.4) | 36.2  (27.9 to 45.2) | 0.21  (0.07 to 0.65) | 1.45  (1.23 to 1.70) | 93.9  (83.1 to 98.7) | 30.8  (22.6 to 39.9) |
| 0.20 | 82.1  (66.5 to 92.5) | 51.2  (42.2 to 60.2) | 0.35  (0.18 to 0.70) | 1.68  (1.33 to 2.12) | 90.3  (80.9 to 96.0) | 34.0  (24.6 to 44.5) |
| **NLR model** | | | | | | |
| 0.1 | 95.0  (82.7 to 99.3) | 29.9  (22.1 to 38.7) | 0.17  (0.04 to 0.68) | 1.35  (1.18 to 1.55) | 95.0  (83.1 to 99.4) | 29.4  (21.6 to 38.1) |
| 0.15 | 74.4  (57.9 to 86.9) | 49.6  (40.6 to 58.6) | 0.52  (0.29 to 0.91) | 1.48  (1.15 to 1.90) | 86.3  (76.3 to 93.2) | 31.2  (21.9 to 41.6) |
| 0.20 | 66.7  (49.8 to 80.9) | 67.7  (58.9 to 75.7) | 0.49  (0.31 to 0.78) | 2.10  (1.48 to 2.89) | 86.9  (78.6 to 92.8) | 38.8  (27.1 to 51.5) |
| **suPAR model** | | | | | | |
| 0.1 | 95.0  (82.7 to 99.4) | 33.1  (24.9 to 41.9) | 0.16  (0.04 to 0.61) | 1.42  (1.23 to 1.63) | 95.4  (84.5 to 99.4) | 30.3  (22.3 to 39.3) |
| 0.15 | 69.2  (52.4 to 82.9) | 55.9  (46.8 to 64.7) | 0.55  (0.34 to 0.90) | 1.57  (1.18 to 2.10) | 85.5  (76.1 to 92.3) | 32.5  (22.7 to 43.7) |
| 0.20 | 56.4  (39.6 to 72.2) | 70.9  (62.2 to 78.6) | 0.62  (0.42 to 0.89) | 1.94  (1.32 to 2.85) | 84.1  (75.8 to 90.4) | 37.3  (25.0 to 50.9) |
| **CRP model** | | | | | | |
| 0.1 | 92.3  (79.1 to 98.4) | 29.9  (22.1 to 38.7) | 0.26  (0.08 to 0.79) | 1.32  (1.14 to 1.52) | 92.7  (80.1 to 98.4) | 28.8  (21.1 to 37.6) |
| 0.15 | 87.2  (72.6 to 95.7) | 44.1  (35.3 to 53.2) | 0.29  (0.13 to 0.67) | 1.56  (1.28 to 1.90) | 91.8  (81.9 to 97.3) | 32.4  (23.6 to 42.2) |
| 0.20 | 69.2  (52.4 to 82.9) | 53.5  (44.5 to 62.4) | 0.57  (0.35 to 0.95) | 1.49  (1.13 to 1.97) | 85.0  (75.3 to 92.0) | 31.4  (21.8 to 42.3) |
| **D-dimer model** | | | | | | |
| 0.1 | 92.3  (79.1 to 98.4) | 29.9  (22.1 to 38.7) | 0.26  (0.08 to 0.79) | 1.32  (1.14 to 1.52) | 92.7  (80.1 to 98.5) | 28.8  (21.1 to 37.6) |
| 0.15 | 82.1  (66.5 to 92.5) | 48.8  (39.8 to 57.8) | 0.37  (0.18 to 0.74) | 1.60  (1.28 to 2.01) | 89.9  (80.2 to 95.8) | 32.9  (23.8 to 43.3) |
| 0.20 | 64.1  (47.2 to 78.8) | 59.8  (50.8 to 68.4) | 0.6  (0.39 to 0.93) | 1.60  (1.16 to 2.19) | 84.4  (75.3 to 91.2) | 32.8  (22.5 to 44.6) |
| **PCT model** | | | | | | |
| 0.1 | 89.7  (75.8 to 97.1) | 25.9  (18.6 to 34.5) | 0.39  (0.15 to 1.05) | 1.21  (1.05 to 1.41) | 89.2  (74.6 to 96.8) | 27.1  (19.7 to 35.7) |
| 0.15 | 76.9  (60.7 to 88.9) | 41.7  (33.1 to 50.8) | 0.55  (0.3 to 1.02) | 1.32  (1.05 to 1.66) | 85.5  (74.2 to 93.1) | 28.9  (20.4 to 38.6) |
| 0.20 | 58.9  (42.1 to 74.4) | 64.6  (55.6 to 72.6) | 0.64  (0.43 to 0.95) | 1.66  (1.17 to 2.37) | 83.7  (78.8 to 90.4) | 33.8  (22.8 to 46.3) |
| **sTREM-1 model** | | | | | | |
| 0.1 | 87.2  (72.6 to 95.7) | 29.1  (21.4 to 37.9) | 0.44  (0.19 to 1.04) | 1.23  (1.04 to 1.45) | 88.1  (74.4 to 96.0) | 27.4  (19.8 to 36.2) |
| 0.15 | 74.4  (57.9 to 86.9) | 46.5  (37.6 to 55.5) | 0.55  (0.31 to 0.97) | 1.36  (1.09 to 1.77) | 85.5  (74.9 to 92.8) | 29.9  (21.0 to 40.0) |
| 0.20 | 61.5  (44.6 to 76.6) | 63.8  (54.8 to 72.1) | 0.60  (0.40 to 0.92) | 1.7  (1.21 to 2.38) | 84.4  (75.5 to 90.9) | 34.3  (23.4 to 46.6) |

**
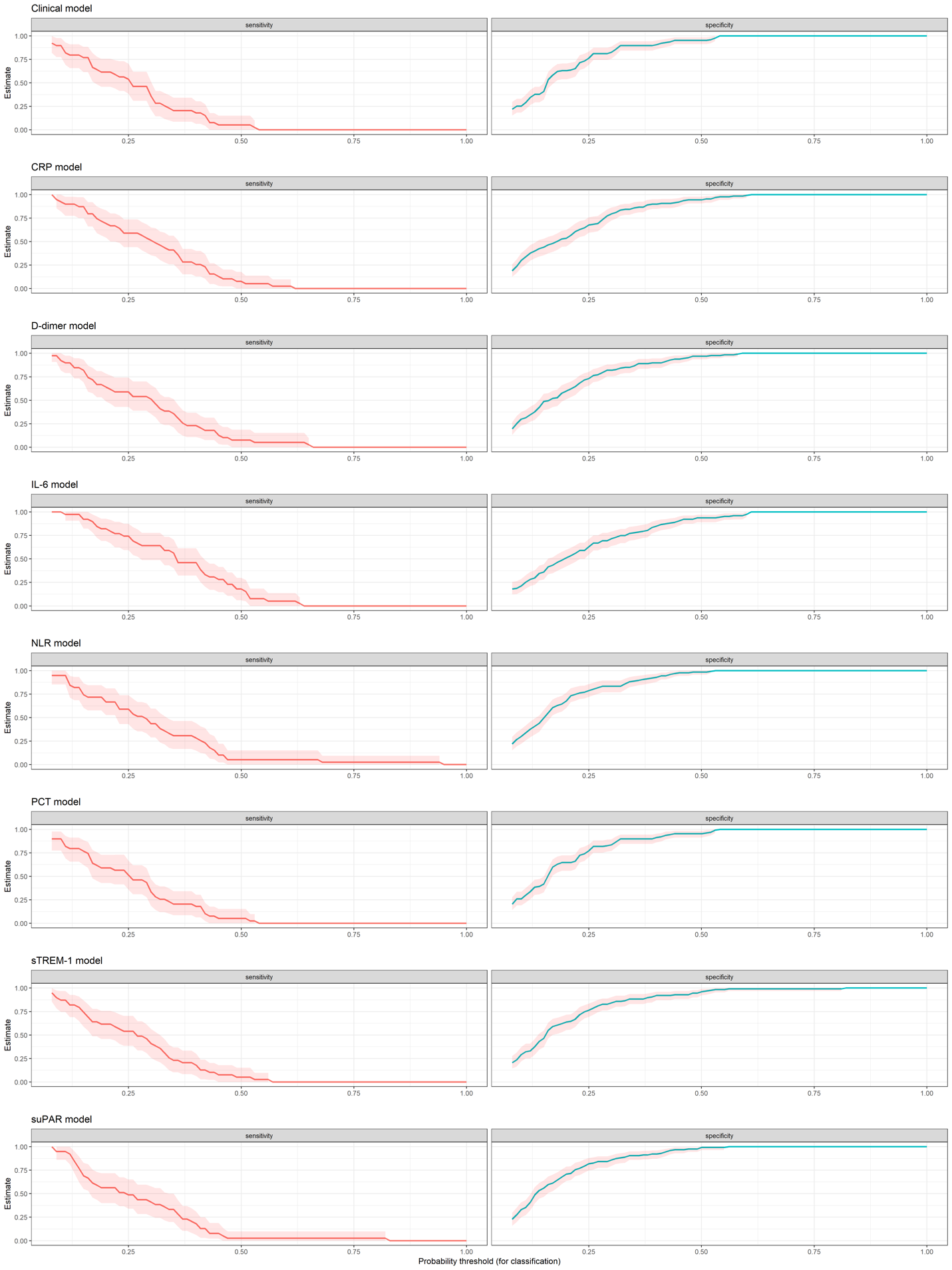
Supplementary Figure 6. Sensitivity and specificity for each model in the validation cohort**. Coloured lines indicate point estimates and pink shaded areas indicate 95% confidence intervals.

**Supplementary Table 7. Association of corticosteroid use with primary outcome.** Corticosteroid use is defined as use of oral or parenteral steroids occurring at least one calendar day prior to developing an oxygen requirement for participants who met the primary outcome.

|  | | **Developed oxygen requirement** | | | **Relative risk**  **(95% CI)** | **p-value** |
| --- | --- | --- | --- | --- | --- | --- |
|  |  | **Yes** | **No** | **TOTAL** |  |  |
| **All participants** | | | | | | |
| **Corticosteroid use** | Yes | 31 | 127 | **158** | 0.90  (0.61 to 1.32) | 0.58 |
|  | No | 58 | 207 | **265** |  |  |
|  | **TOTAL** | **89** | **334** | **423** |  |  |
| **AIIMS participants** | | | | | | |
| **Corticosteroid use** | Yes | 12 | 53 | **65** | 0.73  (0.37 to 1.42) | 0.35 |
|  | No | 15 | 44 | **59** |  |  |
|  | **TOTAL** | **27** | **97** | **124** |  |  |
| **CMC participants** | | | | | | |
| **Corticosteroid use** | Yes | 19 | 74 | **93** | 0.98  (0.60 to 1.58) | 0.93 |
|  | No | 43 | 163 | **206** |  |  |
|  | **TOTAL** | **62** | **237** | **299** |  |  |

**Supplementary Table 8. Candidate predictor variables, stratified by corticosteroid use.** ^*^Missing data: CRP = 8, D-dimer = 3, IL-6 = 2, NLR = 12; PCT = 2; sTREM-1 = 2. Median values (IQR) and p-values for Wilcoxon rank sum tests are reported for continuous variables. Pearson’s Chi-squared test p-values are reported for categorical variables.

| **Candidate predictor** | **Overall**  (n = 423) | **Corticosteroid use** | | **p-value** |
| --- | --- | --- | --- | --- |
|  |  | **No**  (n = 265) | **Yes**  (n = 158) |  |
| Age (years) | 53.0  (41.0 to 62.0) | 53.0  (40.0 to 62.0) | 54.5  (42.0 to 62.0) | 0.4 |
| Male sex | 286 / 423  (68%) | 175 / 265  (66%) | 111 / 158  (70%) | 0.4 |
| Oxygen saturation (%) | 98.0  (96.0 to 99.0) | 98.0  (96.0 to 99.0) | 97.0  (96.0 to 98.0) | 0.017 |
| CRP (mg/l) ^*^ | 36.7  (7.0 to 108.6) | 37.4  (6.8 to 110.3) | 35.5  (7.3 to 101.0) | 0.8 |
| D-dimer (ng/ml) ^*^ | 856.1  (470.8 to 1,522.7) | 846.7  (484.0 to 1,484.5) | 882.1  (458.4 to 1,706.4) | 0.6 |
| IL-6 (pg/ml) ^*^ | 19.8  (6.6 to 47.6) | 21.2  (7.2 to 53.9) | 15.4  (4.8 to 43.2) | 0.042 |
| NLR ^*^ | 3.1  (1.9 to 4.9) | 2.7  (1.7 to 4.3) | 3.7  (2.3 to 6.2) | < 0.001 |
| PCT (ng/ml) ^*^ | 0.1  (0.1 to 0.2) | 0.1  (0.1 to 0.2) | 0.1  (0.1 to 0.2) | 0.8 |
| sTREM-1 (pg/ml) ^*^ | 394.0  (272.0 to 563.0) | 400.0  (269.0 to 563.0) | 381.0  (281.2 to 565.2) | > 0.9 |
| suPAR (ng/ml) | 4.2  (3.1 to 5.7) | 4.1  (3.0 to 5.6) | 4.3  (3.2 to 5.8) | 0.2 |

**Supplementary Table 9. Association of baseline Ct value with primary outcome, stratified by collection technique, PCR platform or both**. Nasopharyngeal swabs (NPS) were collected at CMC and combined nasopharyngeal and oropharyngeal swabs (OPS) were collected at AIIMS. Only swabs collected within the 24 hours prior to recruitment (n = 242/423) are included in this sub-analysis. All swabs were tested using the Cepheid Xpert Xpress SARS-CoV-2, CA at AIIMS. Both the Cepheid Xpert Xpress SARS-CoV-2, CA and Altona RealStar SARS-CoV-2 rRT-PCR, Hamburg were used at CMC. Median values (IQR) and p-values for Wilcoxon rank sum tests are reported.

| **Stratification factor** | **Ct value** | | | **p-value** |
| --- | --- | --- | --- | --- |
|  | **Overall** | **Developed oxygen requirement** | |  |
|  |  | **No** | **Yes** |  |
| All participants (n = 242) | 27.7  (23.1 to 32.7) | 27.4  (22.8 to 33.0) | 28.4  (24.1 to 32.4) | 0.4 |
| **Collection technique (n = 242)** | | | | |
| NPS (n = 118) | 31.5  (26.7 to 35.7) | 31.5  (26.1 to 35.8) | 31.5  (28.0 to 34.6) | 0.9 |
| Combined NPS and OPS (n = 124) | 25.3  (20.5 to 28.5) | 25.5  (20.2 to 28.8) | 24.5  (21.4 to 27.8) | > 0.9 |
| **PCR platform (n = 242)** | | | | |
| Cepheid Xpert Xpress (n = 173) | 26.9  (21.0 to 31.5) | 26.6  (20.7 to 30.9) | 27.6  (24.0 to 32.1) | 0.2 |
| Altona RealStar (n = 69) | 31.6  (27.0 to 35.4) | 31.8  (27.0 to 35.4) | 31.2  (28.0 to 35.4) | 0.6 |
| **PCR platform at CMC site (n = 118)** | | | | |
| Cepheid Xpert Xpress (n = 49) | 31.2  (26.3 to 35.8) | 30.7  (23.2 to 36.1) | 31.9  (28.4 to 33.3) | 0.4 |
| Altona RealStar (n = 69) | 31.6  (27.0 to 35.4) | 31.8  (27.0 to 35.4) | 31.2  (28.0 to 35.4) | 0.6 |

**STROBE Statement—Checklist of items that should be included in reports of *cohort studies***

|  | Item No | Recommendation | Page No |
| --- | --- | --- | --- |
| **Title and abstract** | 1 | (*a*) Indicate the study’s design with a commonly used term in the title or the abstract | 1, 4-5 |
|  |  | (*b*) Provide in the abstract an informative and balanced summary of what was done and what was found | 4-5 |
| Introduction | | | |
| Background/rationale | 2 | Explain the scientific background and rationale for the investigation being reported | 6-7 |
| Objectives | 3 | State specific objectives, including any prespecified hypotheses | 6-7 |
| Methods | | | |
| Study design | 4 | Present key elements of study design early in the paper | 7-8 |
| Setting | 5 | Describe the setting, locations, and relevant dates, including periods of recruitment, exposure, follow-up, and data collection | 7-8 |
| Participants | 6 | (*a*) Give the eligibility criteria, and the sources and methods of selection of participants. Describe methods of follow-up | 7-8 |
|  |  | (*b*) For matched studies, give matching criteria and number of exposed and unexposed | NA |
| Variables | 7 | Clearly define all outcomes, exposures, predictors, potential confounders, and effect modifiers. Give diagnostic criteria, if applicable | 8-10 |
| Data sources/ measurement | 8* | For each variable of interest, give sources of data and details of methods of assessment (measurement). Describe comparability of assessment methods if there is more than one group | 8-10 |
| Bias | 9 | Describe any efforts to address potential sources of bias | 9-10 |
| Study size | 10 | Explain how the study size was arrived at | 10-11 |
| Quantitative variables | 11 | Explain how quantitative variables were handled in the analyses. If applicable, describe which groupings were chosen and why | 11 |
| Statistical methods | 12 | (*a*) Describe all statistical methods, including those used to control for confounding | 11-12 |
|  |  | (*b*) Describe any methods used to examine subgroups and interactions | NA |
|  |  | (*c*) Explain how missing data were addressed | 11 |
|  |  | (*d*) If applicable, explain how loss to follow-up was addressed | 13 |
|  |  | (*e*) Describe any sensitivity analyses | 11 |
| Results | | |  |
| Participants | 13* | (a) Report numbers of individuals at each stage of study—eg numbers potentially eligible, examined for eligibility, confirmed eligible, included in the study, completing follow-up, and analysed | 13 |
|  |  | (b) Give reasons for non-participation at each stage | Fig2 |
|  |  | (c) Consider use of a flow diagram | Fig2 |
| Descriptive data | 14* | (a) Give characteristics of study participants (eg demographic, clinical, social) and information on exposures and potential confounders | 13-14 |
|  |  | (b) Indicate number of participants with missing data for each variable of interest | 13 |
|  |  | (c) Summarise follow-up time (eg, average and total amount) | 13-14 |
| Outcome data | 15* | Report numbers of outcome events or summary measures over time | 13-14 |

| Main results | 16 | (*a*) Give unadjusted estimates and, if applicable, confounder-adjusted estimates and their precision (eg, 95% confidence interval). Make clear which confounders were adjusted for and why they were included | 13-14 |
| --- | --- | --- | --- |
|  |  | (*b*) Report category boundaries when continuous variables were categorized | NA |
|  |  | (*c*) If relevant, consider translating estimates of relative risk into absolute risk for a meaningful time period | NA |
| Other analyses | 17 | Report other analyses done—eg analyses of subgroups and interactions, and sensitivity analyses | 14-16 |
| Discussion | | | |
| Key results | 18 | Summarise key results with reference to study objectives | 16-18, 20-21 |
| Limitations | 19 | Discuss limitations of the study, taking into account sources of potential bias or imprecision. Discuss both direction and magnitude of any potential bias | 18-20 |
| Interpretation | 20 | Give a cautious overall interpretation of results considering objectives, limitations, multiplicity of analyses, results from similar studies, and other relevant evidence | 16, 20-21 |
| Generalisability | 21 | Discuss the generalisability (external validity) of the study results | 15-16, 20-21 |
| Other information | | | |
| Funding | 22 | Give the source of funding and the role of the funders for the present study and, if applicable, for the original study on which the present article is based | 23 |

*Give information separately for exposed and unexposed groups.

**Note:** An Explanation and Elaboration article discusses each checklist item and gives methodological background and published examples of transparent reporting. The STROBE checklist is best used in conjunction with this article (freely available on the Web sites of PLoS Medicine at http://www.plosmedicine.org/, Annals of Internal Medicine at http://www.annals.org/, and Epidemiology at http://www.epidem.com/). Information on the STROBE Initiative is available at <http://www.strobe-statement.org>.

| **Section/Topic** | **Item** |  | **Checklist Item** | **Page** |
| --- | --- | --- | --- | --- |
| **Title and abstract** | | | | |
| Title | 1 | D;V | Identify the study as developing and/or validating a multivariable prediction model, the target population, and the outcome to be predicted. | 1 |
| Abstract | 2 | D;V | Provide a summary of objectives, study design, setting, participants, sample size, predictors, outcome, statistical analysis, results, and conclusions. | 4 |
| **Introduction** | | | | |
| Background and objectives | 3a | D;V | Explain the medical context (including whether diagnostic or prognostic) and rationale for developing or validating the multivariable prediction model, including references to existing models. | 6-7 |
|  | 3b | D;V | Specify the objectives, including whether the study describes the development or validation of the model or both. | 6-7 |
| **Methods** | | | | |
| Source of data | 4a | D;V | Describe the study design or source of data (e.g., randomized trial, cohort, or registry data), separately for the development and validation data sets, if applicable. | 7-8 |
|  | 4b | D;V | Specify the key study dates, including start of accrual; end of accrual; and, if applicable, end of follow-up. | 13 |
| Participants | 5a | D;V | Specify key elements of the study setting (e.g., primary care, secondary care, general population) including number and location of centres. | 7 |
|  | 5b | D;V | Describe eligibility criteria for participants. | 7-8 |
|  | 5c | D;V | Give details of treatments received, if relevant. | NA |
| Outcome | 6a | D;V | Clearly define the outcome that is predicted by the prediction model, including how and when assessed. | 8-9 |
|  | 6b | D;V | Report any actions to blind assessment of the outcome to be predicted. | 9 |
| Predictors | 7a | D;V | Clearly define all predictors used in developing or validating the multivariable prediction model, including how and when they were measured. | 9-10 |
|  | 7b | D;V | Report any actions to blind assessment of predictors for the outcome and other predictors. | 10 |
| Sample size | 8 | D;V | Explain how the study size was arrived at. | 10-11 |
| Missing data | 9 | D;V | Describe how missing data were handled (e.g., complete-case analysis, single imputation, multiple imputation) with details of any imputation method. | 11 |
| Statistical analysis methods | 10a | D | Describe how predictors were handled in the analyses. | 11 |
|  | 10b | D | Specify type of model, all model-building procedures (including any predictor selection), and method for internal validation. | 11 |
|  | 10c | V | For validation, describe how the predictions were calculated. | 12 |
|  | 10d | D;V | Specify all measures used to assess model performance and, if relevant, to compare multiple models. | 11-12 |
|  | 10e | V | Describe any model updating (e.g., recalibration) arising from the validation, if done. | NA |
| Risk groups | 11 | D;V | Provide details on how risk groups were created, if done. | NA |
| Development vs. validation | 12 | V | For validation, identify any differences from the development data in setting, eligibility criteria, outcome, and predictors. | NA |
| **Results** | | | | |
| Participants | 13a | D;V | Describe the flow of participants through the study, including the number of participants with and without the outcome and, if applicable, a summary of the follow-up time. A diagram may be helpful. | 13 |
|  | 13b | D;V | Describe the characteristics of the participants (basic demographics, clinical features, available predictors), including the number of participants with missing data for predictors and outcome. | 13-14 |
|  | 13c | V | For validation, show a comparison with the development data of the distribution of important variables (demographics, predictors and outcome). | 13-14 |
| Model development | 14a | D | Specify the number of participants and outcome events in each analysis. | 13-14 |
|  | 14b | D | If done, report the unadjusted association between each candidate predictor and outcome. | 14 |
| Model specification | 15a | D | Present the full prediction model to allow predictions for individuals (i.e., all regression coefficients, and model intercept or baseline survival at a given time point). | 14 |
|  | 15b | D | Explain how to the use the prediction model. | 14-15 |
| Model performance | 16 | D;V | Report performance measures (with CIs) for the prediction model. | 14-15 |
| Model-updating | 17 | V | If done, report the results from any model updating (i.e., model specification, model performance). | NA |
| **Discussion** | | | | |
| Limitations | 18 | D;V | Discuss any limitations of the study (such as nonrepresentative sample, few events per predictor, missing data). | 18-20 |
| Interpretation | 19a | V | For validation, discuss the results with reference to performance in the development data, and any other validation data. | NA |
|  | 19b | D;V | Give an overall interpretation of the results, considering objectives, limitations, results from similar studies, and other relevant evidence. | 16, 20-21 |
| Implications | 20 | D;V | Discuss the potential clinical use of the model and implications for future research. | 16, 20-21 |
| **Other information** | | | | |
| Supplementary information | 21 | D;V | Provide information about the availability of supplementary resources, such as study protocol, Web calculator, and data sets. | 12-13, 22 |
| Funding | 22 | D;V | Give the source of funding and the role of the funders for the present study. | 23 |

**TRIPOD Checklist: Prediction Model Development and Validation**

1. Patients presenting without virologically-confirmed SARS-CoV-2 but meeting all other eligibility criteria will be recruited. They will be removed from the study if they are subsequently confirmed to be negative for SARS-CoV-2 via RT-PCR. [↑](#footnote-ref-1)
2. SpO_2_ ≤ 93% OR respiratory rate > 30 breaths per minute OR clinical decision to give supplemental oxygen. [↑](#footnote-ref-2)
